# Detecting gene breakpoints in noisy genome sequences using position-annotated colored de-Bruijn graphs

**DOI:** 10.1186/s12859-023-05371-4

**Published:** 2023-06-05

**Authors:** Lisa Fiedler, Matthias Bernt, Martin Middendorf, Peter F. Stadler

**Affiliations:** 1 Department of Computer Science, University Leipzig, Augustusplatz 10-11, 04109 Leipzig, Germany; 2 Helmholtz Centre for Environmental Research -UFZ, Permoserstraße 15, 04318 Leipzig, Germany; 3 Bioinformatics Group, Department of Computer Science, and Interdisciplinary Center for Bioinformatics, Universität Leipzig, Härtelstraße 16–18, 04107 Leipzig, Germany; 4 Max Planck Institute for Mathematics in the Sciences, Inselstraße 22, 04109 Leipzig, Germany; 5 Department of Theoretical Chemistry, University of Vienna, Währinger Straße 17, 1090 Vienna, Austria; 6 Facultad de Ciencias, Universidad National de Colombia, Sede Bogotá, Ciudad Universitaria, 111321 Bogotá, D.C., Colombia; 7 Santa Fe Institute, 1399 Hyde Park Rd., Santa Fe, NM 87501 USA

**Keywords:** Gene breakpoints, de-Bruijn graph, Genome, Mitochondria

## Abstract

**Background:**

Identifying the locations of gene breakpoints between species of different taxonomic groups can provide useful insights into the underlying evolutionary processes. Given the exact locations of their genes, the breakpoints can be computed without much effort. However, often, existing gene annotations are erroneous, or only nucleotide sequences are available. Especially in mitochondrial genomes, high variations in gene orders are usually accompanied by a high degree of sequence inconsistencies. This makes accurately locating breakpoints in mitogenomic nucleotide sequences a challenging task.

**Results:**

This contribution presents a novel method for detecting gene breakpoints in the nucleotide sequences of complete mitochondrial genomes, taking into account possible high substitution rates. The method is implemented in the software package DeBBI. DeBBI allows to analyze transposition- and inversion-based breakpoints independently and uses a parallel program design, allowing to make use of modern multi-processor systems. Extensive tests on synthetic data sets, covering a broad range of sequence dissimilarities and different numbers of introduced breakpoints, demonstrate DeBBI ’s ability to produce accurate results. Case studies using species of various taxonomic groups further show DeBBI ’s applicability to real-life data. While (some) multiple sequence alignment tools can also be used for the task at hand, we demonstrate that especially gene breaks between short, poorly conserved tRNA genes can be detected more frequently with the proposed approach.

**Conclusion:**

The proposed method constructs a position-annotated de-Bruijn graph of the input sequences. Using a heuristic algorithm, this graph is searched for particular structures, called bulges, which may be associated with the breakpoint locations. Despite the large size of these structures, the algorithm only requires a small number of graph traversal steps.

## Introduction

Breakpoints in genomic sequences are locations where related sequences fail to be collinear and consequently cannot be globally aligned. Breakpoints thus also delimit structural variants (SVs), i.e., genomic variations between individuals of a certain species. SVs include deletions, insertions, duplications, and inversions. A large number of computational tools, usually referred to as *variant callers*, have been developed aiming at the identification of breakpoint locations resulting from SVs in high-throughput sequencing data. Most of these methods require a reference genome, see e.g. [1–5]. Some other approaches work with short substrings of length *k*, called *k*-mers, but rely on known variant or reference *k*-mers [6–8]. Meanwhile, callers for direct comparison of sequence reads have also been proposed [9, 10]. Moreover, some specialized tools have been suggested for identifying SV inversions [11], copy number variations [12], and SVs in long-read sequencing data [13]. Variant callers are designed to operate on closely related sequences and have applications, in particular, in the investigation of disease mechanisms and cancer research [14–17].

Here, we are interested in detecting breakpoint locations that appear at larger evolutionary time scales and are associated with the divergence of the genomes of distinct species due to changes in gene content and arrangement of gene orders. A long-standing question for mitochondrial genomes is whether rearrangements are created by a duplication/loss, a cut-and-paste mechanism, or a mixture of both. Thus the ability to detect/predict such breakpoint regions is important for studying rearrangement mechanisms with methods like [18]. In addition, we show that a breakpoint-based approach will supplement purely similarity/local-colinearity-based approaches.

If both, the gene orders and the genes’ exact positions are known, the breakpoints between the genes can easily be computed [19, 20]. In general, however, only the nucleotide sequences are available, and annotations of gene positions are approximate at best. To detect them nevertheless, one option is to first employ a sequence aligner that can consider gene rearrangements. Breakpoints can then be computed by identifying alignment blocks that are consecutive in one genome but not in another.

Theoretical concepts of a more direct approach are presented by Lin et al. [21], showing that the de-Bruijn graph is essentially identical to the breakpoint graph. The latter has been the most frequently employed data structure for breakpoint analyses in the last decades. Lin et al. show that in the de-Bruijn, breakpoints between genes correspond to specific structures. These so-called *bulges* are common paths that branch into two separate paths, which join again at a different location. However, so far, no method has been proposed to computationally identify such bulges and handle artifacts that arise from the sequence dissimilarities when sequences of distinct species are compared. Usually, large variations in gene orders are accompanied by large substitution rates, in particular, in mitochondrial genomes so that sequence inconsistencies cannot be neglected to study gene rearrangements therein.

Several practical applications of bulges in de-Bruijn graphs have been described in the literature. These include variant callers such as GRIDDS [10], TakeABreak [5], and Cortex [6]. Another use case has been the removal of sequencing errors, as implemented in the Velvet [22] software suite. In both applications, short bulges with similar branch lengths are of interest. This permits employing graph-traversal-based search strategies, such as Velvet’s tour bus algorithm. However, for the identification of the here-considered breakpoints between genes, up to genome-length-sized bulges are relevant. This renders such approaches inapplicable, even for moderately sized genomes.

Here, we present a novel approach optimized for identifying breakpoints between genes from nucleotide sequences of complete mitochondrial genomes. The method uses the input sequences to construct a position-annotated colored de-Bruijn graph. Genome rearrangements are frequent in mitogenomes, rendering them particularly interesting for breakpoint analysis. At the same time, rearranged genes in mitochondrial sequences are particularly prone to suffer from high sequence mutation rates  [23–25], generally to a much larger extent than in nuclear genomes. To this end, the concept of a breakpoint bulge, as introduced in [21], is extended to take sequence dissimilarities into account, and a heuristic algorithm is presented to identify such bulges with only a small number of graph-traversal steps. The vast majority of gene rearrangements in mitogenomes is caused by transpositions or tandem duplication random loss (TDRL) [26] events (see, e.g., [23, 27]), which is why they will be the main objective of this work. In each such case, genes are dislocated only within the same strand. These types of rearrangement will thus be referred to as *dislocations*. Gene *inversions*, which move genes to the opposite strand, or inverse transpositions (i.e., a combination of transposition and inversion), occur much more rarely (cf. [23]). In the “Methods” section below, we discuss dislocations in full detail. To keep the presentation concise, inversions will only be sketched briefly.

The proposed methods are implemented in a software package called DeBBI (*De*-*B*ruijn graph-based tool for *B*reakpoint *I*dentification). DeBBI comprises two independent programs for identifying breakpoints caused by gene dislocations and gene inversions. This allows for studying both types of breakpoints separately. Both programs may be run in arbitrary order without affecting the produced results. DeBBI features a parallel program design to take advantage of modern multi-processor systems. To minimize the necessary amount of manual interaction, DeBBI provides a routine for the automated computation of the $(k+1)$-mer size of the de-Bruijn graph and features well-tested default settings for the remaining program parameters.

In this study, we consider both synthetic, as well as, real-life data sets of different taxonomic groups. Using a simple model of sequence evolution, the synthetic data sets are generated to comprise sequence inconsistencies of various degrees and gene arrangements of different deviations. Exact breakpoint locations can be computed in this case and used as ground truth data. To assess the result accuracy of the real genome sequences, gene annotations generated by the mitochondrial gene annotation tool MITOS2  [28] are used to compute putative breakpoint locations.

To our knowledge, there is so far no method specifically dedicated to the task of identifying gene breakpoints in genomes from nucleotide sequences. Unfortunately, variant callers (such as GRIDDS, TakeABreak, or Cortex), which are available in large numbers, are not applicable. To give some examples: They work with sequencing reads rather than complete genome sequences; they often employ certain assembly-related statistics, which do not apply in this case; they are designed to operate locally to detect structural variations between individuals of a population, rather than to discover breaks between heavily rearranged genes in more distantly related species.

For this reason, we use the breakpoint predictions that can be determined from the sequence alignment blocks of a genome aligner (see above) for a comparative analysis. As a representative of this class of tools, we employ progressiveMauve [29, 30]. This is one of the most influential and well-established methods in this field. Moreover, it computes the large majority of the required parameters from the input data and otherwise provides well-established settings. Contrarily, in other alignment tools (which like progressiveMauve can take into account rearranged sequence segments) certain properties, which substantially impact the result quality, need to be specified manually. This renders large-scale automatic evaluations infeasible. For example, in Gecko [31], an alignment similarity parameter, which can attain all values between 1 and 100, must be provided. Another example is CHROMEISTER, where the *k*-mer length needs to be set. In the supplementary material (Additional file 1), we conduct some additional experiments with Gecko and CHROMEISTER on selected real genome sequences that have also been analyzed with progressiveMauve, empirically determining suitable parameter settings for each case.

## Methods

In this section, we assume that genomes are circular, as is the case for most mitochondrial sequences. The handling of linear genomes requires only minor changes. These are described in Additional file 1: Section *Handling linear genomes*.

### Dislocation breakpoints

**Fig. 1:**
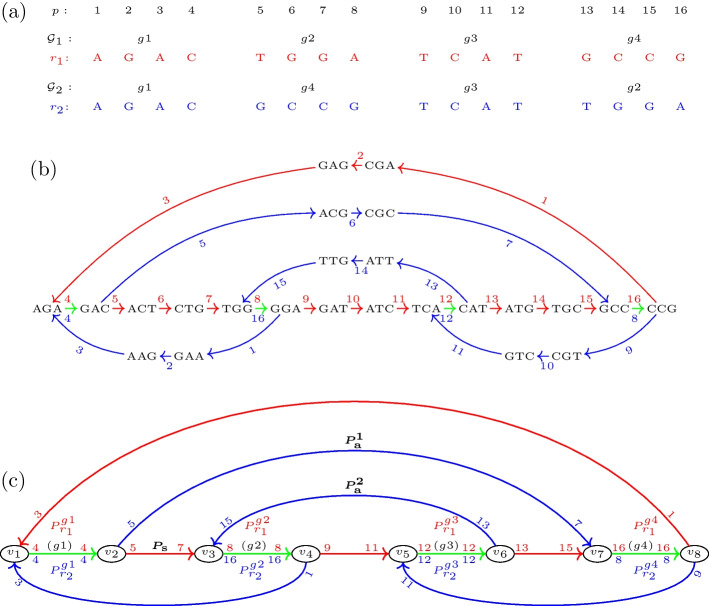
De-Bruijn graph of two genomes $r_1$ and $r_2$ with dislocation breakpoints. **a** Genome $r_1$ with gene sequence $\mathcal {G}_1=(g_1,g_2,g_3,g_4)$ and genome $r_2$ with gene sequence $\mathcal {G}_2=(g_1,g_4,g_3,g_2)$. **b** The de-Bruijn graph of both genomes for $k=3$. An edge is colored red if it corresponds to genome $r_1$, blue if it corresponds to $r_2$, and green if it corresponds to both genomes. Moreover, it is annotated with its position in the genome, where position labels of $r_1$ are red and annotated above the edge, and position labels of $r_2$ are blue and annotated below the edge. **c** The de-Bruijn graph with all condensed single and 2-color paths. Each of the 2-color paths $\{P_{r_1}^{gi},P_{r_2}^{gi}\}$ is annotated with the associated maximal synteny block (MSB) (*gi*). There are eight breakpoints between both genomes, resulting in eight breakpoint bulges (BBs) in the graph. Exemplarily, the defining single-color paths of the BB for $(g_1,g_2)_{1,2}$ are labeled by $P_{\text{s}}$, $P_{\text{a}}^1$ and $P_{\text{a}}^2$ and highlighted in bold font. Path $P_{\text{s}}$ constitutes the single-color branch in this bulge. $P_{\text{a}}^1$ and $P_{\text{a}}^2$ form the beginning and end of the color-alternating branch, respectively

For a more comprehensible presentation, we use the toy example scenario of Fig. 1, which we refer to throughout this section.

The gene order of a genome is the sequence of its genes in the order in which they occur (duplicate genes may be contained). Denote the gene orders of two species by $\mathcal {G}_1$ and $\mathcal {G}_2$. An adjacent pair of genes $(g_i,g_j)$ of $\mathcal {G}_2$ is called a dislocation breakpoint of $\mathcal {G}_2$ with respect to $\mathcal {G}_1$ if both genes are also contained in but do not appear consecutively in that order in $\mathcal {G}_1$. For better readability, $(g_i,g_j)_{1,2}$ is used if $(g_i,g_j)$ is a breakpoint in $\mathcal {G}_1$ with respect to $\mathcal {G}_2$, and $(g_i,g_j)_{2,1}$ in the opposite situation. Thus in the example setting (Fig. 1), the dislocation breakpoints are given as $(g_1,g_2)_{1,2},(g_2,g_3)_{1,2}$, $(g_3,g_4)_{1,2}$, $(g_4,g_1)_{1,2}$, $(g_1,g_4)_{2,1},(g_4,g_3)_{2,1}$, $(g_3,g_2)_{2,1}$, and $(g_2,g_1)_{2,1}$.

A breakpoint $(g_i,g_j)_{1,2}$ means that there is some gene $g_m\ne g_j$ succeeding $g_i$ and some gene $g_n\ne g_i$ preceding $g_j$ in $\mathcal {G}_2$. Every breakpoint $(g_i,g_j)_{1,2}$ is thus accompanied by two breakpoints $(g_i,g_m)_{2,1}$ and $(g_n,g_j)_{2,1}$, which will be referred to as *entangled* breakpoints from now on. For instance, in the toy example the entangled breakpoints of $(g_1,g_2)_{1,2}$ are $(g_1,g_4)_{2,1}$ and $(g_3,g_2)_{2,1}.$

Regions of DNA between genomes that share a common order of *n* homologous genes $g_i,g_{i+1},\dots ,g_{n}$ are called *synteny blocks* $(g_i,g_{i+1},\dots ,g_{n})$. A synteny block is called maximal (MSB) if it is not contained in any other synteny block. For the gene orders in the toy example, the MSBs consist only of the individual genes, i.e., $(g_1)$, $(g_2),(g_3)$, and $(g_4)$.

#### Dislocation breakpoints in the de-Bruijn graph

We consider the de-Bruijn graph over a set of circular input mitogenomes *G* as a directed multigraph. Its vertex set *V* comprises all *k*-mers in *G*, i.e., substrings of length *k* in *G*. These can be obtained by sliding a window of length *k* over the genome sequences. The edges are 4-tuples of the form $(v,v',r,p)$ and correspond to the substring *s* of length $(k+1)$ in genome $r\in G$ with end-position *p*. The two vertices *v* and $v'$ are length *k* prefix and suffix of *s*, respectively. Each mitogenome *r* of length |*r*| therefore contributes exactly |*r*| edges to the de-Bruijn graph. The position *p* refers to arbitrary but fixed linear coordinates on *r*. The annotated de-Bruijn graph of the toy example is illustrated in Fig. 1b. To improve the clarity of the presentation, edges are colored to refer to different individual (red and blue) or multiple (green) genomes.

**Fig. 2:**
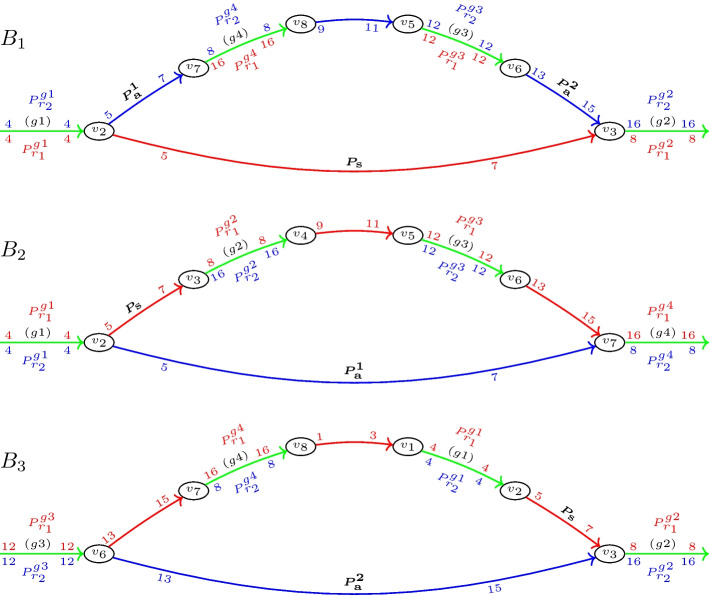
Detailed view of three breakpoint bulges (BBs) $B_1$, $B_2$, and $B_3$ of the de-Bruijn graph shown in Fig. 1, respectively corresponding to breakpoints $(g_1,g_2)_{1,2}$, $(g_1,g_4)_{2,1}$, and $(g_3,g_2)_{2,1}$. Single-color branches are shown on the bottom, and color-alternating branches on the top of each bulge. Path $P_S$, which constitutes the single-color branch in $B_1$, is part of the color-alternating branch in $B_2$ and $B_3$. Path $P_a^{1}$, which forms the beginning of the color-alternating branch in $B_1$, constitutes the single-color branch in $B_2$ and path $P_a^{2}$, which forms the end of the color-alternating branch in $B_1$, constitutes the single-color branch in $B_3$

Every dislocation breakpoint $(g_i,g_j)_{1,2}$ between two genomes $r_1, r_2 \in G$ translates to a special type of bulge between $g_i$ and $g_j$, referred to as *breakpoint bulge* (BB), in the de-Bruijn graph. A bulge is formed by two edge-disjoint paths, termed *branches*, that originate from a common vertex $v_b$ and meet again in another vertex $v_m\ne v_b$. In a BB, one of the branches is short and of a single color, while the other branch is long and color-alternating.

We make use of the following notion to formally describe BBs. Define a *single-color path* $P_r=(\mathcal {V},\mathcal {E}_r)$ of genome $r\in G$ and length $|P_r|=n$ as a sequence $\mathcal {V}$ of *n* distinct vertices $(v_1,v_2,\dots ,v_n)$. These are connected by a sequence $\mathcal {E}_r$ of $n-1$ edges $((v_1,v_2,r,p_1),(v_2,v_3,r,p_2),\dots ,(v_{n-1},v_n,r,p_{n-1}))$ with $p_{i+1}={{\,\text{succ}\,}}(p_i,1) \, \forall i \in \{1,\dots ,n-2\}$. Here, ${{\,\text{succ}\,}}(p,l)$ is the $l^{\text{th}}$ position succeeding *p*. Analogously, ${{\,\text{pred}\,}}(p,l)$ will be used to refer to the $l^{\text{th}}$ position preceding *p*. Since the positions of adjacent edges in $P_r$ are also adjacent in the de-Bruijn graph, $P_r$ is already uniquely defined by the first and last position of edges in $\mathcal {E}_r$ and can thus be *condensed* to the ordered tuple $(r,p_1,p_{n-1})$. We call a collection of *m* single-color paths $\{P_{r_1},P_{r_2},\cdots , P_{r_m}\}$ where $\mathcal {V}(P_{r_1})=\mathcal {V}(P_{r_2})=\dots =\mathcal {V}(P_{r_m})$ and $r_i \ne r_j$ for $i,j \in \{1,2,\dots ,m\}$ and $i \ne j$ a *m*-color path. The *m* single-color paths $P_{r_1},P_{r_2},\cdots , P_{r_m}$ thus describe identical sequence segments in genomes $r_1,r_2,\cdots ,r_m$. Figure 1c shows the de-Bruijn graph of Fig. 1b of our toy example with annotated condensed single-color and 2-color paths.

Now reconsider the three entangled breakpoints $(g_1,g_2)_{1,2}$, $(g_1,g_4)_{2,1}$ and $(g_3,g_2)_{2,1}$. For two genes $g_i$ and $g_j$ that are consecutive in a genome, denote by *transition* $(k+1)$-mers all $(k+1)$-mers of this genome where the prefix is part of the encoding sequence of $g_i$, and the suffix is part of the encoding sequence of $g_j$. Because of the different order of genes of $r_1$ and $r_2$, all of $r_1$’s transition $(k+1)$-mers between $g_1$ and $g_2$, are not transition $(k+1)$-mers of $r_2$ (the two genes are not consecutive in $r_2$). Likewise, $r_2$’s transition $(k+1)$-mers between $g_1$ and $g_4$ and $r_2$’s transition $(k+1)$-mers between $g_3$ and $g_2$ are not transition $(k+1)$-mers of $r_1$.

This results in three single-color paths in the de-Bruijn graph (cf. Fig. 1c): $P_{\text{s}}=(r_1,5,7)$, which connects 2-color path $\{P_{r_1}^{g1},P_{r_2}^{g1}\}$ of MSB $(g_1)$ with 2-color path $\{P_{r_1}^{g2},P_{r_2}^{g2}\}$ of MSB $(g_2)$, $P_{\text{a}}^1=(r_2,5,7)$, which connects 2-color path $\{P_{r_1}^{g1},P_{r_2}^{g1}\}$ with 2-color path $\{P_{r_1}^{g4},P_{r_2}^{g4}\}$ of MSB $(g_4)$, and $P_{\text{a}}^2=(r_2,13,15)$ which connects 2-color path $\{P_{r_1}^{g3},P_{r_2}^{g3}\}$ of MSB $(g_3)$ with the 2-color path $\{P_{r_1}^{g2},P_{r_2}^{g2}\}$. In the BB of $(g_1,g_2)_{1,2}$, the first of these paths $P_{\text{s}}$ constitutes the single-color branch of the bulge. On the color-alternating branch, paths $P_{\text{a}}^1$ and $P_{\text{a}}^2$ form the beginning and end, respectively. The three paths thus *define* the BB. However, path $P_{\text{a}}^1$ also constitutes the single-color branch and $P_{\text{s}}$ forms the beginning of the color-alternating branch in the BB of $(g_1,g_4)_{2,1}$, while path $P_{\text{a}}^2$ constitutes the single-color branch and $P_{\text{s}}$ forms the end of the color-alternating branch in the BB of $(g_3,g_2)_{2,1}$. That is, every BB $B_1$ shares single-color paths with the two BBs $B_2$ and $B_3$ of its entangled breakpoints. More particularly, paths that are located on the single-color branch in $B_1$ are located on the color-alternating branch in $B_2$ and $B_3$, while paths that are located on the color-alternating branch in $B_1$ are located on the single-color branch in $B_2$ and $B_3$. To better recognize this relation, Fig. 2 shows the three bulges individually.

**Fig. 3:**
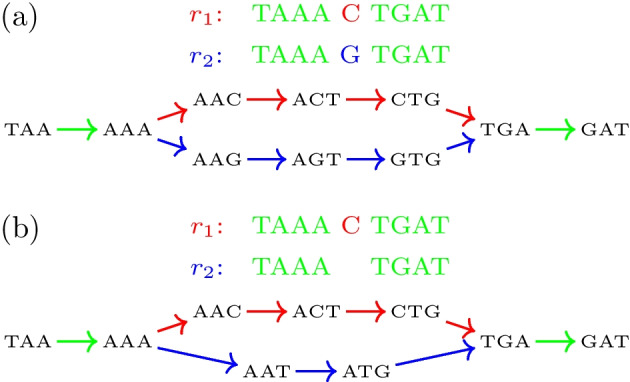
Inconsistency bulges caused by single nucleotide variations between genomes $r_1$ and $r_2$ in a de-Bruijn graph with $k=3$. **a** A single nucleotide polymorphism causes $k+1=4$ unmatched edges, resulting in an inconsistency bulge with two single-color branches of length $k+1=4$. **b** A single nucleotide deletion in $r_2$ causes $k+1=4$ unmatched edges in $r_1$ and $k=3$ unmatched edges in $r_2$, resulting in an inconsistency bulge with two single-color branches of length $k+1=4$ and $k=3$, respectively

Small sequence dissimilarities result in short *inconsistency bulges* of similar branch lengths, deteriorating the clear structure of the BBs described above. Figure 3 illustrates two such bulges. Removing these inconsistencies from the graph can destroy meaningful biological information. Moreover, it is an expensive operation as the graph must usually be modified to a large extent. It is also not clear how to resolve them in many cases, i.e., which path should be preferred over the other. As reported by various studies, a higher variation in the gene order of mitogenomes corresponds with higher substitution rates and thus promotes a high degree of sequence inconsistency [23–25]. In particular, rearranged mitochondrial tRNA genes are often poorly conserved, while at the same time being rearranged most frequently among all mitochondrial genes. Thus, for the gene breakpoint detection in mitogenomes it is of great importance to take such events into account.

#### Identification of dislocation breakpoints in noisy sequences

**Fig. 4:**
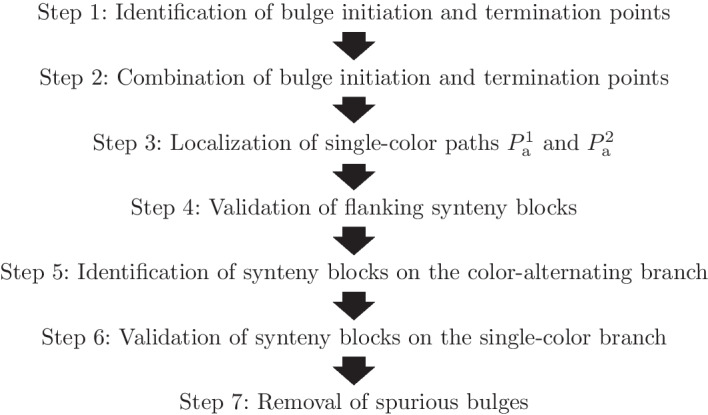
Workflow for the identification of dislocation breakpoints

The workflow of our method is summarized in Fig. 4. A major problem in our setting is that rearranged mitochondrial sequences are “noisy” due to the high degree of sequence dissimilarities in the input (see section above). We, therefore, start from suitable pairs of branching paths whose combination *might* give rise to BBs. Steps 1 and 2 in our workflow thus create a set of BB candidates. The single-color paths on the branches of the BB candidates are subsequently determined by exploiting the threefold entangled nature of dislocation breakpoints (Step 3). Thereafter, the candidates are checked for MSBs on the branches and bulge flanks (Steps 4–6). Finally, a cleansing routine removes remaining spurious candidates (Step 7). The individual Steps are described below.

**Fig. 5:**
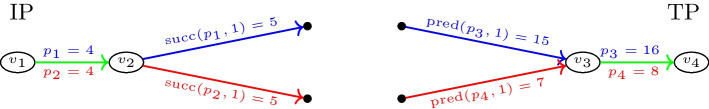
A pair of bulge initiation (IP) and termination points (TP) for BB $B_1$ of the toy example (cf. Figs. 1, 2). For both IPs and TPs, the four vertices are distinct and the positions of adjacent edges of the same genome are succeeding, i.e., have a distance of one

*Step 1* Initially, the graph is examined for two types of possible branch points: Initiation points (IP), where a 2-color path splits into two single-color paths, and termination points (TP), where two single-color paths merge into a 2-color path. The precise definition of both branch points is illustrated in Fig. 5 using the toy example.

*Step 2* IPs are locations in the graph where a path common to two genomes diverges, and if it later re-converges in a TP, a bulge is formed. Hence, each combination of an IP with a TP of the same two genomes $r_1$ and $r_2$ yields two candidates for BBs, one where the putative breakpoint is in $r_1$ with respect to $r_2$ and the other one with the roles of $r_1$ and $r_2$ exchanged. In the IP-TP pair of Fig. 5, the first candidate would be the correct one ($r_1$ with respect to $r_2$) and correspond to BB $B_1$ of Fig. 2. For this candidate, the single-color branch can already directly be determined as $P_{\text{s}}=(r_1,5,7)$ from the IP-TP combination. On the color-alternating branch, so far only the first and last edges are specified by $(v_2,\bullet ,r_2,5)$ and $(\bullet ,v_3,r_2,15)$, respectively, where $\bullet$ is a placeholder for an irrelevant value.

In a true BB, the color-alternating branch contains at least one MSB composed of at least one gene. Thus, its length must at least be of the size $|g_{\text{min}}|$ of the shortest gene $g_{\text{min}}$ that is typically present in the class of species under consideration. The single-color branch, on the other hand, does not contain any synteny blocks and hence is notably shorter. Its precise length depends mainly on the degree of conservation between the genomes under consideration and the presence and size of intergenic regions but generally is in the order of *k*. Details may be found in Additional file 1: Section *Branch lengths*. These two conditions on the branch lengths are used as a rough initial filter to sort out combined IP-TP pairs that cannot result in BBs. For the above-considered IP-TP pair, this filter would already remove the incorrect second candidate.

*Step 3* As discussed in Section “Dislocation breakpoints in the de-Bruijn graph”, every true BB shares two paths each with its entangled BBs. For $B_1$ of the toy example, these are $P_s$ and one of paths $P_{\text{a}}^1$ or $P_{\text{a}}^2$ (cf. Fig. 2). This relation is now used to identify the missing edges of paths $P_{\text{a}}^1$ and $P_{\text{a}}^2$ on the color-alternating branch of the $B_1$ candidate, eliminating the need for expensive graph traversals. In detail, to complete $P_{\text{a}}^1$, whose first edge $(v_2,\bullet ,r_2,5)$ is already known, other IP-TP combinations are searched whose single-color branch is a single-color path of $r_2$ and starts with position 5. Analogously, $P_{\text{a}}^2$, whose last edge $(\bullet ,v_3,r_2,15)$ is already known, can be completed by searching for IP-TP combinations whose single-color branch again is a single-color path of $r_2$ but now ends with position 15. These conditions are met by the candidates for bulge $B_2$ and $B_3$, respectively. Consequently, $P_{\text{a}}^1$ can be set to the single-color branch $(r_2,5,7)$ of $B_2$ and $P_{\text{a}}^2$ can be set to the single-color branch $(r_1,13,15)$ of $B_3$. If there were no two such IP-TP combinations to complete the paths for the $B_1$ candidate, this candidate could not be a true BB and could thus be removed. Hence, this step also serves as an additional filter for random candidates.

Sequence inconsistencies may lead to additional IP-TP combinations that satisfy the above conditions and could thus be used for the path completion of the considered candidate. In this case, the routine selects the pair of IP-TP combinations and, thereby, completes paths $P_{\text{a}}^1$ and $P_{\text{a}}^2$ so that among all possible options both paths are furthest apart. The rationale is that for every other choice, at least one of the paths contains 2-color path segments (otherwise, there would not be a combination of paths with a greater distance), which are already part of the MSBs on the color-alternating branch enclosed by $P_{\text{a}}^1$ and $P_{\text{a}}^2$.

*Step 4* Every true BB is flanked by one MSB on each bulge end, e.g., $(g_1)$ and $(g_2)$ for BB $B_1$. The subsequences corresponding to each of the flanking 2-color paths of a BB candidate should hence be similar. Since a MSB must consist of at least one gene of size $|g_{\text{min}}|$ (cf. Step 2), we consider only subpaths of size $\rho = |g_{\text{min}}|+\epsilon$ and thus also subsequences of this length. Here, $\epsilon$ is a small value for which in the case of mitogenomes $\epsilon =20$ proved to be a good choice (cf. Additional file 1: Section *Local sequence alignments* for details).

To assess the similarity between the subsequences, we employ a banded local sequence alignment with affine gap costs (cf. Additional file 1: Section *Local sequence alignments* for parameter settings) and accept alignments below a specified *E*-value threshold where at least $n^{\text{match}}$ nucleotides match perfectly. The latter also implicitly determines the minimum length the alignments must have. Candidates, where at least one of the alignments does not fulfill these conditions are discarded. These constraints will also be used for the alignments of later steps.

**Fig. 6:**
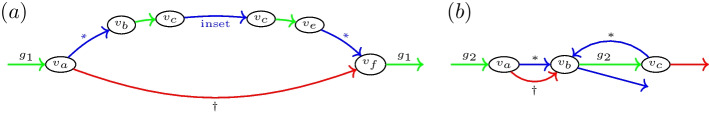
Two spurious breakpoint bulge (BB) candidates. In each case, the defining path of the single-color branch is annotated by $\dagger$. The two defining paths of the color-alternating branch are annotated by $*$. **a** Spurious candidate between $v_a$ and $v_f$ caused by a longer inset of nucleotides in gene $g_1$ in $r_2$ (blue). The green edges on the upper bulge branch indicate only short random mappings of both genomes, no actual homologous sequence segments. **b** Spurious candidate between $v_a$ and $v_b$. There is a homologous region between $v_b$ and $v_c$ on the color-alternating branch of this candidate, but it is equal to the homologous region on the right flank, i.e., both overlap to $100\%$. The candidate is thus not shaped like a bulge

*Step 5* The homologous regions identified at the two flanks of the candidate BBs could still be part of the same MSB, which is interrupted by a series of sequence inconsistencies. This could be due to a longer inset of nucleotides (or a series of deletions) in one of the genomes, such as shown in Fig. 6a. Here, genome $r_2$ features such an inset in $g_1$, which is not present in $r_1$. While Step 1 removes most of such cases, a non-negligible number may be retained, in particular, if the considered species are only moderately or poorly conserved. To eliminate them, the color-alternating branches of the candidates are examined for homologous regions, which are missing in the above scenario.

To see how this can be done, reconsider BB $B_1$ of the toy example (cf. Figs. 1, 2). In this case, there are two homologous regions, MSBs $(g_4)$ and $(g_3)$, on the color-alternating branch. These correspond to the two 2-color paths $\{P_{r_1}^{g_4},P_{r_2}^{g_4}\}$ and $\{P_{r_1}^{g_3},P_{r_2}^{g_3}\}$, respectively. Since $\{P_{r_1}^{g_4},P_{r_2}^{g_4}\}$ starts directly after $P_{\text{a}}^1$, the first edge of its $r_2$ component $P_{r_2}^{g_4}$ is the positional direct successor to the last edge on $P_{\text{a}}^1$, i.e., $(v_7,\bullet ,r_2,{{\,\text{succ}\,}}(7,1)=8)$. Likewise, 2-color path $\{P_{r_1}^{g_3},P_{r_2}^{g_3}\}$ ends directly before $P_{\text{a}}^2$. Thus, the last edge of its $r_2$ component $P_{r_2}^{g_3}$ is the positional direct predecessor to the first edge on $P_{\text{a}}^2$, i.e., $(\bullet ,v_6,r_2,{{\,\text{pred}\,}}(13,1)=12)$.

Like in Step 4, only the first $\rho$ vertices of the first and the last $\rho$ vertices of the second putative 2-color paths are considered. For the $B_1$ candidate, the single-color paths $P_{\text{a}}^1$ and $P_{\text{a}}^2$ are already known. The $r_2$ components of the 2-color paths can thus already be determined as $(r_2,8,{{\,\text{succ}\,}}(8,\rho ))$ and $(r_2,{{\,\text{pred}\,}}(12,\rho ),12)$. To find out whether the corresponding $r_1$ components exist, we search for alignment anchors on the $r_2$ paths by combining consecutive $(k+1)$-mer matches of $r_1$. To this end, a local sorting strategy is employed, again eliminating the need for graph traversals. This is done by Algorithm 1 described in Additional file 1: Section *Alignment anchor detection*.

A point mutation distinguishing $r_1$ and $r_2$ generally results in $k+1$ unmatched edges in the de-Bruijn graph (cf. Fig. 3). As a consequence, there may be multiple separate alignment anchors that are output by Algorithm 1, which are actually part of the same homologous region. Considered on their own, these individual paths may not be sufficient indicators for homology because the path length is too short. Therefore, we chain alignment anchors as long as possible and subsequently extend them linearly to cover the $r_2$ components of the 2-color paths. Lastly, the resulting subsequences are aligned. The detailed steps are explained in Additional file 1: Section *Alignment anchor chaining* using Algorithm 2.

BB candidates are discarded if only low-quality alignments could be found. Otherwise, all good-quality alignments are regarded as homologous regions. As in the case depicted in Fig. 6b, these regions might still coincide with the regions that have been identified at the bulge flanks in Step 5. To detect such spurious candidates, we evaluate the pairwise overlap between these regions. A small overlap is tolerated to account for possible random $(k+1)$-mer mappings. Based on manual experimentation with a large number of different mitochondrial genomes, a threshold of $10\%$ was found to be a good choice to sort out the large majority of such cases while still not incorrectly retaining candidates where these regions coincide.

*Step 6* While the single-color branches are generally only approximately *k* nucleotides long, poorly conserved species or long intergenic regions may also cause longer single-color branch lengths (confer Additional file 1: Section *Branch lengths*). Such branches would, however, still not contain homologous regions. Candidate BBs with single-color branch lengths of at least $n^{\text{match}}$ (recall trustworthy alignments must involve at least that many perfectly matching nucleotides) are thus assessed to see whether they contain such regions. To this end, the previous step is repeated in a similar manner with the single-color branch as input to Algorithm 1. In contrast to Step 5, candidate BBs are discarded if an alignment of sufficient quality is encountered.

**Fig. 7:**
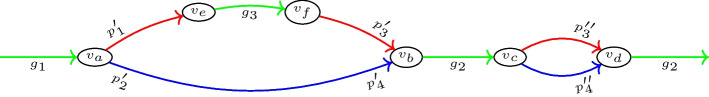
Spurious “shifted ” breakpoint bulge (BB) candidate. In addition to the correct BB candidate *B* between $v_a$ and $v_b$, a second spurious candidate $B'$ between $v_a$ and $v_d$ exists for breakpoint $(g_1,g_2)_{2,1}$. This second candidate is caused by an inconsistency bulge close to the end of gene $g_2$ between $v_c$ and $v_d$

*Step 7* Finally, the last step removes “shifted” BB candidates. Consider Fig. 7: Sequence inconsistencies within gene $g_2$ result in an inconsistency bulge (cf. Fig. 3) between vertices $v_c$ and $v_d$. This may result in two candidates for breakpoint $(g_1,g_2)_{2,1}$. One of them is the correct BB *B* between vertices $v_a$ and $v_b$. The second one is a spurious candidate $B'$ between vertices $v_a$ and $v_d$. There are several criteria that must be met to cause such a scenario. For example, the inconsistency region must be close to the start of $g_2$. The complete list of conditions and their detailed explanation is compiled in Additional file 1: Section *Shifted breakpoint bulge candidates*.

To identify such spurious candidates, we apply the agglomerative clustering routine AGNES (agglomerative nested clustering) [32] to the remaining BB candidates. This clustering routine builds a hierarchy, i.e., a tree, of clusters by greedily merging data points, candidate BBs in this case, in a bottom-up fashion. To decide which clusters to combine, a similarity measure needs to be specified. Here, this is the maximum pairwise distance between the related position predictions at the bulge ends of two candidates. In the previous example, this would be the distances between $p_3'$ and $p_3''$, as well as $p_4'$ and $p_4''$. Clusters are not joined if the candidates have different genomes annotated on their branches. When no more clusters can be merged, the created tree is cut at $\rho$. This yields single-point clusters for unambiguous candidates and multi-point clusters if shifted bulges and thus ambiguities for the same breakpoint event exist. To remove the spurious candidates in the multi-point clusters, only the cluster with the shortest bulge branches is selected, e.g., bulge *B* in the above example.

**Fig. 8:**
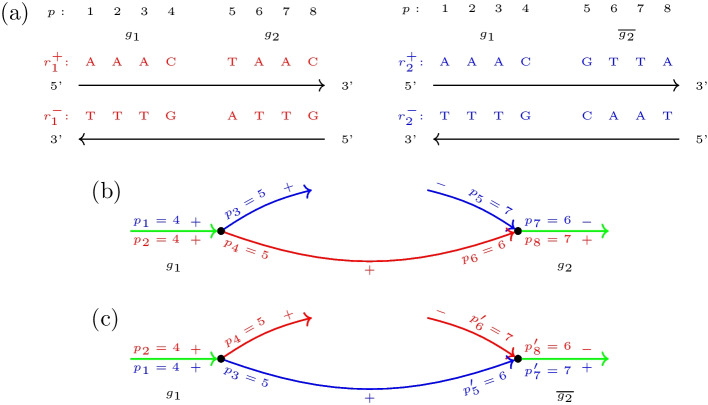
“Broken” bulges caused by a gene inversion. **a** While gene $g_1$ is encoded on the plus strand in both genomes $r_1$ and $r_2$, gene $g_2$ is located on opposite strands, i.e., on the plus strand in $r_1$ and minus strand in $r_2$. The reverse complement $\overline{g_2}$ is located on the respective other strand. Note the opposing reading directions of both strands indicated by the arrows. In the two-stranded de-Bruijn graph ($k=2$), this results in one bulge **b** for $g_2$ and one bulge **c** for $\overline{g_2}$. Note that due to the redundancy introduced by adding $(k+1)$-mers of both strands, there is an additional pair of bulges with negative-strand $(k+1)$-mer matches on the left bulge flanks in the graph. These bulges are ignored because they do not yield any further information

### Gene inversion breakpoints

Inversions are rearrangements that invert a continuous part of the chromosome. As a consequence, a sequence of genes, which was located on the affected segment on one strand, will be located on the opposite strand afterward. On the sequence level, this means that the corresponding encoding sequence $s=s_1s_2\dots s_n$ is replaced by its reverse complement $\overline{s}=s^{C}_ns^{C}_{n-1}\dots s^{C}_1$ on the original strand, while $\overline{s}$ is replaced by *s* on the opposite strand (both in 5’ to 3’ direction). Here, $x^{C}$ of a nucleotide *x* is its complementary nucleotide, e.g., $A^{C}=T$ and $C^{C}=G$. To follow along, we make use of a second toy example, which is shown in Fig. 8. Here, gene $g_2=TAAC$, which is located on the positive strand (reading direction left to right) in $r_1$ has undergone an inversion in $r_2$. Consequently, $\overline{g}_2=GTTA$ is located on the positive strand in $r_2$, while $g_2$ is encoded on the negative strand (reading direction right to left).

To identify such segments in the de-Bruijn graph, the graph introduced in the “Dislocation breakpoints in the de-Bruijn graph” section must be modified to also include the $(k+1)$-mers of the negative strands. To this end, every edge $(v,v',r,p)$ in the previous graph is replaced by a new edge $(v,v',r,p,+)$. In addition, the edge $(\overline{v},\overline{v}',r,p-k,-)$, representing the corresponding sequence segment on the complementary strand, is included. Note that consecutive $(k+1)$-mers on the negative strand have decreasing position annotations. This is due to opposing reading directions of both strands and the convention that genome positions are specified with respect to the positive strand.

In this two-stranded de-Bruijn graph, gene inversions are expressed by 2-color paths, which are formed by $(k+1)$-mer matches of opposite strands. In the above toy example, $r_1$’s $(k+1)$-mers $(TA,AA,r_1,7,+)$ and $(AA,AC,r_1,8,+)$ of $g_2$, which are located on the positive strand, match with $r_2$’s $(k+1)$-mers $(TA,AA,r_2,6,-)$ and $(AA,AC,r_2,5,-)$, respectively, which are located on the negative strand. For the reverse complement $\overline{g}_2=GTTA$, by symmetry, $r_1$’s $(k+1)$-mers $(GT,TT,r_1,6,-)$ and $(TT,TA,r_1,5,-)$ match with $r_2$’s $(k+1)$-mers $(GT,TT,r_1,7,+)$ and $(TT,TA,r_1,8,+)$, respectively. As a result, two “broken” bulges (Fig. 8b, c) arise in the graph. In these bulges, positions ${{\,\text{pred}\,}}(p_6,k-1)=5$ to ${{\,\text{succ}\,}}(p_6',1)=8$ confine the sequence segment of $g_2$ in $r_1$ on the positive strand, while positions ${{\,\text{succ}\,}}(p_5,1)=8$ to ${{\,\text{pred}\,}}(p_5',k-1)=5$ confine this segment in $r_2$ on the negative strand, both in reading direction 5’ to 3’ (i.e., left to right on the plus and right to left on the minus strand). We call these segments *inverted sequence blocks* (IBs).

Therefore, to identify IBs, the graph is searched for pairs of broken bulges, creating a set of candidates. In contrast to the toy example, the gene content is generally not perfectly conserved. The IBs are hence not necessarily of the same size. To account for this, a small relative deviation of

$$\begin{aligned} \frac{|\Delta (p_6,p_6')-\Delta (p_5',p_5)|}{\min \{ \Delta (p_6,p_6'),\Delta (p_5',p_5)\}} \le \delta \end{aligned}$$

is permitted, where $\Delta (p,p')$ is the distance from *p* to $p'$. For similar reasons as in Step 5 of the previous section, $\delta = 10\%$ was found to be a good choice.

The thus obtained sequence segments are subsequently examined for homology using a global sequence alignment. Candidates with alignment scores *a*, so that

$$\begin{aligned} \frac{a-a_{\text{min}}}{a_{\text{max}}-a_{\text{min}} }\ge \tilde{a} \end{aligned}$$

are accepted as IBs, where $a_{\text{min}}$ and $a_{\text{max}}$ are the minimum and maximum possible alignment scores, respectively.

### Determination of the (*k* + 1)-mer size

The probably most important parameter that must be set at the beginning of the proposed approach is the $(k+1)$-mer size of the de-Bruijn graph. Generally, an approximate exponential growth of the runtimes can be expected for decreasing values of *k*. Depending on the considered mitogenomes, this may cause extremely long runtimes from a certain point on. However, this is not necessarily a loss, since a smaller value of *k* does not inevitably improve the accuracy of the results. On the contrary, from a certain point on, the result quality will be impaired. This is because lowering the $(k+1)$-mer size also increases the number of random matches between unrelated sequence segments. Thus a compromise between a too-large value, concealing many sequence similarities among the genomes, and a too-small value, cluttering the graph with many random matches must be found.

It is often difficult to decide which value best satisfies these requirements for a given set of input genomes. Therefore, while this value can be specified manually as a runtime parameter, we also provide an automatic computation routine, which is employed if no parameter is passed to the program.

To this end, we determine the $(k+1)$-mer size as the minimum value so that the $(k+1)$-mer repeat rate for each sequence in the de-Bruijn graph is at most 0.15. Here, this rate is defined as $1-($number of unique $(k+1)$-mer$\hbox {s}/$ number of all $(k+1)$-mer$\hbox {s})$. Manual experimentation with many different mitochondrial sequences suggested that this is generally a good choice to both keep the required runtimes at bay and produce good-quality predictions. An evaluation of experiments with several selected mitogenomes can be found in Additional file 1: Section *Impacts of the* $(k+1)$-mer *size on the runtime and result accuracy*.

### Data sets

#### Simulated data sets

Simulated data sets were constructed from a simple model of sequence evolution along a rooted tree. For each experiment, ten direct child sequences were generated from this parental sequence by introducing nucleotide substitutions using the HYK model of nucleotide substitution implemented in the simulation package Seq-gen [33]. The transition/transversion rate was set to 3.0, and an equal substitution rate $r_{\text{sub}}$ was applied to all children. This way, the ratio of base pairs shared between every pair of child sequences $s_1$ and $s_2$ of length $|s_1|$ and $|s_2|$ is approximately $1-2r_{\text{sub}}/\min \{|s_1|,|s_2|\}$. By using substitution rates of $r_{\text{sub}}\in \{1,3,5,7.5\}$, four child sets $C_1,C_3,C_5$, and $,C_{7.5}$ were evolved, covering low, intermediate, and high levels of sequence similarity, respectively. We used a metazoan mitogenome of typical composition (i.e., encoding for 13 proteins, 22 tRNAs, and 2 rRNAs) as a parental sequence at the root to simulate data that closely resembles mitogenomes.

**Table 1:** Number of breakpoints contained in each of the sets $R_{n_{\text{ra}}}$, $n_{\text{ra}} \in \{1,3,5,10\}$

Set	Gene dislocations per gene order	Total number of breakpoints	Mean number of breakpoints per gene order	Mean number of breakpoints per pair of gene orders
$R_1$	1	518	52	12
$R_3$	3	1350	135	30
$R_5$	5	1762	176	39
$R_{10}$	10	2658	266	59

Shown is the number of genes that have been dislocated in each of the ten gene orders in the respective set (col. 2), the total number of breakpoints between all 45 pairwise combinations of the ten gene orders in this set (col. 3), the mean number of breakpoints that one gene order has with any of the other nine gene orders in this set (col. 4), and the mean number of breakpoints between one pair of gene orders in this set (col. 5)

Dislocation breakpoints were modeled by moving different randomly selected genes in the gene order of the parental sequence to other locations. One set $R_1$, one set $R_3$, one set $R_5$, and one set $R_{10}$, each composed of ten gene orders, were created by changing the location of one gene, three genes, five genes, and ten genes each, respectively. To this end, first, a gene and, subsequently, a new location was selected randomly, taking care to restrict dislocations to regions outside the bounds of other gene encoding segments. This process was repeated once, three times, five times, and ten times to create one gene order in set $R_1$, $R_3$, $R_5$, and $R_{10}$, respectively, starting over for the next gene order until all ten gene orders were generated for the respective set. Table 1 shows the resulting number of breakpoints between the gene orders in the sets. Note that this number is twice that of the breakpoint distance [19, 20] commonly used in rearrangement studies. Each set $R_{n_{\text{ra}}}$, $n_{\text{ra}} \in \{1,3,5,10\}$ was applied once to each set $C_{r_{\text{sub}}}$, $r_{\text{sub}}\in \{1,3,5,7.5\}$. Given two sets $R_{n_{\text{ra}}}$ and $C_{r_{\text{sub}}}$, the ten rearranged gene orders of $R_{n_{\text{ra}}}$ were randomly allocated to the ten sequences in $C_{r_{\text{sub}}}$, modifying the sequences accordingly by moving the affected encoding subsequences to their new locations, thus creating 16 sets for the synthetic dislocation experiments, composed of ten sequences each.

In order to produce a test set for identifying gene inversions, seven subsets $C_{r_{\text{sub}}}'$, each consisting of three randomly selected sequences of the original set $C_{r_{\text{sub}}}$, were used. In each such subset, two inversions of randomly selected tRNA genes were introduced to one sequence, two inversions of randomly selected proteins to another sequence, while one sequence was left unaltered, creating a total of eight inversion blocks (IB) per subset.

#### Real genome data sets

We consider three real genome data sets for gene dislocation experiments and one real genome data set for gene inversion experiments. Each data set is composed of complete mitogenomes contained in RefSeq89. The RefSeq database [34] is the most comprehensive and up-to-date resource for curated, non-redundant mitochondrial genomes along with their annotation.

### Implementation

The proposed approach is released as a free open-source software package called DeBBI. DeBBI is implemented in Apache Spark [35] using its Java API. Spark is a large-scale data processing analytics engine, which provides implicit (data) parallelism for multi-processor systems or computing clusters. This facilitates that large parts of the graph can simultaneously be examined for breakpoint bulges. Moreover, efficient group, sort, and other operations can be achieved.

## Results

We introduce here a new de-Bruijn graph based method to identify gene breakpoints in mitochondrial genomes with substantial sequence divergence. To our knowledge, DeBBI is the first dedicated tool for this task. In the following, we compare DeBBI with progressiveMauve [30], a widely used state-of-the-art tool for multiple genome alignment that considers rearrangement events. While designed to derive alignments, it can also be used for the detection of gene breakpoints by identifying alignment blocks that are consecutive in one genome, but not in another. Both progressiveMauve and DeBBI require only nucleotide sequences as input, thus affording a fair comparison.

### Benchmarking procedure and parameter settings

In all conducted experiments, progressiveMauve’s runtime parameters were set as suggested by [30]. For DeBBI, the $(k+1)$-mer size was computed automatically, as described in the “Determination of the (*k* + 1)-mer size” section. This resulted in a value of $k=10$ for all but one experiment, where a value of $k=12$ was determined. For the alignments in the dislocation breakpoint routine, we use an *E*-value threshold of $10^{-5}$ and require a minimum number of $n^{\text{match}}=20$ perfectly matching nucleotides. For the relative alignment score $\tilde{a}$ of the inversion breakpoint routine, we use a value of 0.8. From manual experimentations, these parameters have been found to generally produce good-quality predictions. By using them as default settings, we hope to relieve a user from the cumbersome empirical work of choosing suitable parameter settings. However, advanced users may provide other values.

If gene annotations for the considered genomes are available, the breakpoint locations can in principle be computed from the positions of two genes that are consecutive in one of the genomes but not in another one. However, existing annotations of real mitochondrial sequences have limited accuracy of annotated gene ends, may contain overlapping genes, and in some cases, annotations may be inaccurate as far as the identity of the genes is concerned. It is thus not possible to determine the precise breakpoint locations from this data by automated means. For large-scale evaluations of the result accuracy, we, therefore, use simulated data where precise gene annotations are known so that the precise expected breakpoint locations can be computed. To showcase DeBBI ’s applicability to real-life data, selected real genome sequences, where the result quality is assessed manually, are considered thereafter.

### Simulated data

Note that, in order to facilitate the computation of the precise breakpoint locations in the simulated data sets, only nucleotide substitutions are considered in the simulated sequences. The position of the gene boundaries is thus not altered before the gene blocks are rearranged/inverted in the sequences. Contrarily, if insertions and deletions had been incorporated additionally, these events might have affected the gene boundaries and consequently the breakpoint locations themselves.

**Fig. 9:**
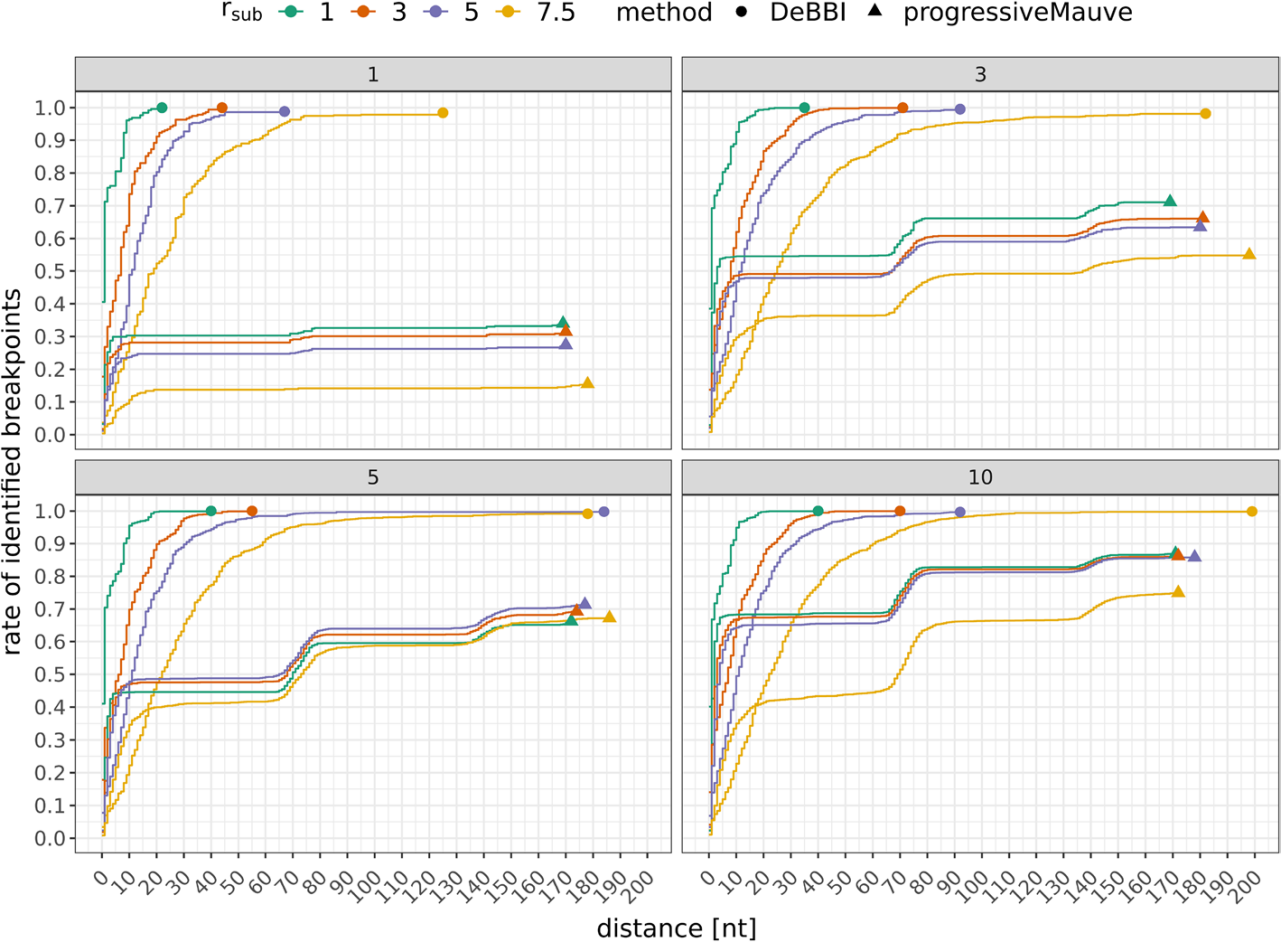
Empirical distribution functions (EDFs) of the synthetic dislocation experiments. The four plots summarize the result quality for the experiments with one, three, five, and ten gene dislocations per genome and different substitution rates $r_{\text{sub}}$. The best rate of correctly identified breakpoints, i.e., the end of the EDFs is marked by a circle or triangle for DeBBI and progressiveMauve, respectively

#### Gene dislocations

Figure 9 shows the *empirical distribution functions* (EDFs) of distances of breakpoints identified by DeBBI and progressiveMauve to the exactly computed breakpoints of the synthetic dislocation experiments, normalized to the total number of exactly computed breakpoints per experiment. That is, for each curve, the value at a specified distance describes the rate of breakpoints found with positional error up to this distance. For the DeBBI predictions, the curves are very similar in all of the four plots, indicating that DeBBI is robust with respect to variations in the number of breakpoints. Up to and including $r_{\text{sub}}=5$, DeBBI identified nearly all breakpoints within a maximum distance of $50{{\,\mathrm{\, \text{nt}}\,}}$. Even for the highest substitution rate of 7.5, more than $90\%$ of the exactly computed breakpoints were detected within a distance of at most $70{{\,\mathrm{\, \text{nt}}\,}}$.

In general, progressiveMauve ’s curves assume lower values than the corresponding EDF of DeBBI for all distances. progressiveMauve tries to find collinear blocks between the input sequences. Manual inspection showed that its attempt to keep these blocks as large as possible often results in either aligning sequence segments even though these contain a breakpoint or not identifying the collinear blocks flanking a breakpoint because the blocks are too small. This effect is particularly prevalent for breakpoints between tRNA genes. Mitochondrial tRNAs are about $70{{\,\mathrm{\, \text{nt}}\,}}$ long and make up almost $60\%$ of the genes in the mitogenomes used in the experiments (typical composition). This explains the plateau-like shape and abrupt increase in the identified breakpoint rate at increments of approximately $70{{\,\mathrm{\, \text{nt}}\,}}$.

**Table 2:** Number of correctly identified inversion blocks by DeBBI and progressiveMauve in the synthetic data sets

$r_{\text{sub}}$	1	3	5	7.5
progressiveMauve	5	5	6	6
DeBBI	8	8	7	6

#### Gene inversions

Table 2 summarizes the number of correctly identified IBs found by progressiveMauve and DeBBI. IB predictions are considered correct if they share at least $75\%$ of their positions with the corresponding true IB. As shown in the table, DeBBI correctly identified at least as many IBs as progressiveMauve in each experiment. Up to and including $r_\text{sub}=3$, DeBBI even detected all eight IBs correctly.

### Case studies on real genome sequences

For the computation of putative breakpoint locations for the real genome data sets, we employ the gene predictions generated by MITOS2  [28]. Using these annotations instead of the annotations in the RefSeq database serves to reduce misannotations and naming inconsistencies. For the same reason, only protein-coding genes, tRNAs, and rRNAs are considered; non-coding regions such as replication origins are disregarded.

**Fig. 10:**
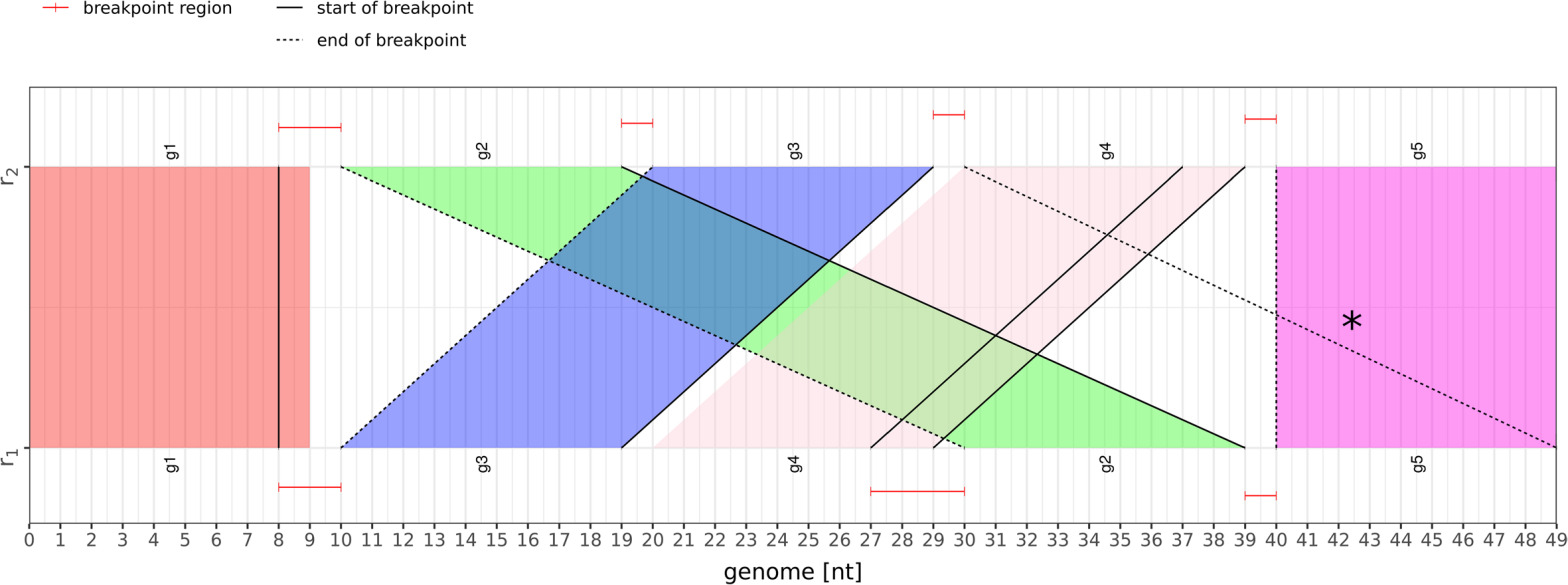
Example breakpoint plot for two genomes $r_1$ and $r_2$. Sequence segments encoding for the same gene are given the same color and annotated with the same gene name $g_1,g_2,g_3,g_4$, or $g_5$. The red intervals on the top and bottom of the figure show the predicted locations for a breakpoint. Black lines connect these locations to the identified related site in the other genome. For better readability, these lines are solid for the start and dashed for the end of a breakpoint. For example, the first red interval from positions eight to ten on the top left corresponds to breakpoint $(g_1,g_2)_{1,2}$. The connecting lines end at positions 8 and 30 in the bottom genome. Ideally, these lines should coincide with the corresponding boundary of the colored gene blocks for $g_1$ and $g_2$. Here, this is only true for the $g_2$ boundary. Thus, there is a distance of one of the predicted to the putative breakpoint location. There are no deviations for breakpoints $(g_2,g_3)_{1,2},(g_4,g_5)_{1,2},$ and $(g_2,g_5)_{2,1}$. Connecting lines that are not parallel to any of the colored areas indicate that the corresponding predicted locations cannot be allocated to any of the putative breakpoints. This is the case for the dashed line marked by an asterisk. The corresponding prediction is incorrect since there is no breakpoint between genes $g_3$ and $g_4$

To also visually assess the quality of the predicted breakpoint locations in the context of the MITOS2 gene annotations, a plot referred to as *breakpoint plot* was designed, showing which areas of the genomes are involved in a predicted breakpoint with respect to the gene annotations. Figure 10 illustrates how to interpret this plot with a simple example.

To estimate the degree of sequence similarity for the considered mitogenomes, we determined the pairwise $(k+1)$-mer *match rates* and *inversion match rates* for the dislocation and inversion experiments, respectively. The match rates are defined as the number of distinct $(k+1)$-mer matches between two genomes divided by the total number of $(k+1)$-mers of the shorter of both genomes. It should be noted, however, that this is only a rough measure, as different genes may be conserved to a varying degree, while these rates are evaluated for the complete genome as a whole. The inversion match rates consider only the $(k+1)$-mer matches of opposing strands. As a reference, we also determined these rates for the simulated sequences. They are shown in Additional file 1: Table S1.

#### Gene dislocations

Each of the three gene dislocation studies was performed using two mitogenomes. The species of the different experiments were selected to cover the three major bilaterian groups Vertebrata, Spiralia, and Arthropoda, represented by two Sillago, two Decapodiformes, and two Nematocera species, respectively (cf. Table 3). Additional file 1: Figure S9 shows the taxonomic tree for the considered species.

**Table 3:** Real genome gene dislocation case studies

Accession IDs		lca	Match rate	Putative breakpoints
NC_030373	NC_025935	Sillago	0.26	6
NC_007894	NC_009690	Decapodiformes	0.12	52
NC_016202	NC_16173	Nematocera	0.18	40

Shown are the accession IDs, the lowest common ancestor (lca), the $(k+1)$-mer match rates, and the number of putative breakpoints for each of the experiments

**Fig. 11:**
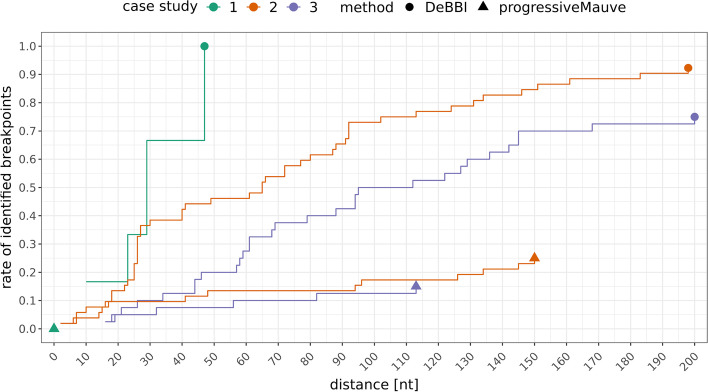
Empirical distribution functions (EDFs) of the real genome dislocation experiments. Putative breakpoints are computed from gene annotations generated by MITOS2. The best rate of correctly identified breakpoints, i.e., the end of the EDFs, is marked by a circle or triangle for DeBBI and progressiveMauve, respectively. The additional breakpoints found within the non-coding regions are not taken into account as they cannot be associated with any of the putative breakpoints, which only consider breaks between gene-encoding regions

The first experiment concerns closely related bony fish, *Sillago aeolus* and *Sillago sinica*. The two mitogenomes differ only by the exchanged position of two consecutive tRNA genes (cf. Additional file 1: Section *Gene orders of dislocation case study 1* and Additional file 1: Figure S11). This results in six putative breakpoints (cf. Table 3). DeBBI successfully identified all of them with deviations of less than $50{{\,\mathrm{\, \text{nt}}\,}}$, as can be seen in Fig. 11, which shows the EDFs of the DeBBI and progressiveMauve predictions with respect to the putative breakpoint locations computed with MITOS2. The corresponding breakpoint plot is shown in Additional file 1: Figure S16.

**Fig. 12:**
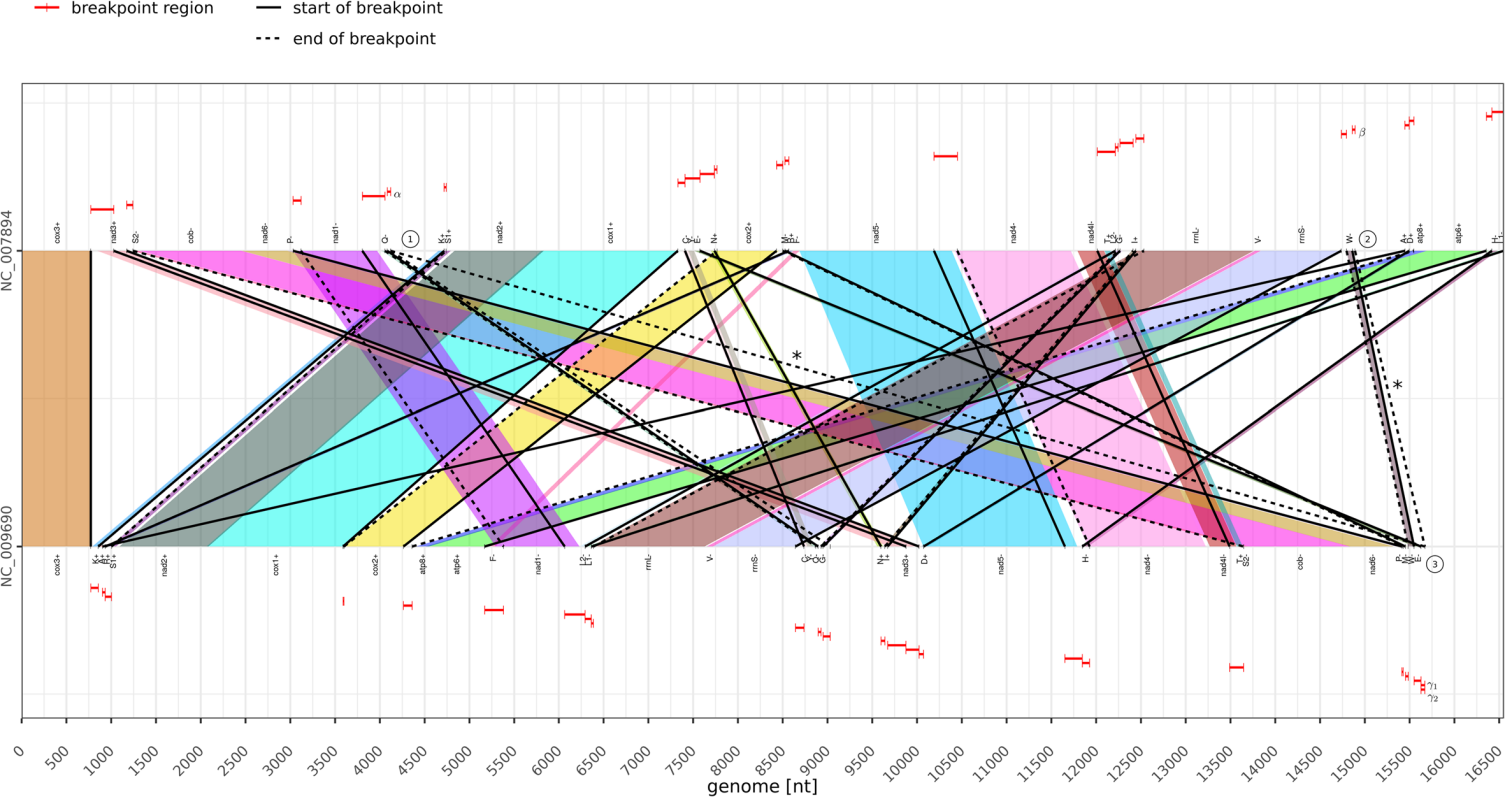
Breakpoint plot of the Decapodiforme data set (second case study)

The two Decapodiformes of the second case study feature an exceptionally high number of 52 putative breakpoints (cf. Table 3), of which DeBBI detects the great majority with $d_{\text{max}}\le 200$ (cf. Fig. 11). Moreover, both genomes contain an unusually high number (for metazoan mitogenomes) of comparably long non-coding regions (cf. Fig. 12). Such long non-coding segments were described previously for Cephalopoda and Decapodiformes [36], containing identical sequence elements with possible transcription and/or replication functions. DeBBI indeed identified highly similar sequence segments in three non-coding regions of the here-considered genomes. In the corresponding breakpoint plot of Fig. 12, this leads to two anti-parallel lines, marked by asterisks, which connect these regions (annotated by encircled numbers one to three). Note that this is different from the anti-parallel line of the example scenario shown in Fig. 10, which connects two different gene encoding regions $g_4$ and $g_5$, thus causing an incorrect breakpoint prediction, as opposed to non-coding segments in this case, which do not cause erroneous predictions. The four resulting additional breakpoint regions are annotated by the Greek letters $\alpha ,\beta ,\gamma _1$, and $\gamma _2$, where $\gamma _1$ and $\gamma _2$ correspond to the same sequence segment in the bottom sequence, once associated with region one and once associated with region two in the top sequence.

**Fig. 13:**
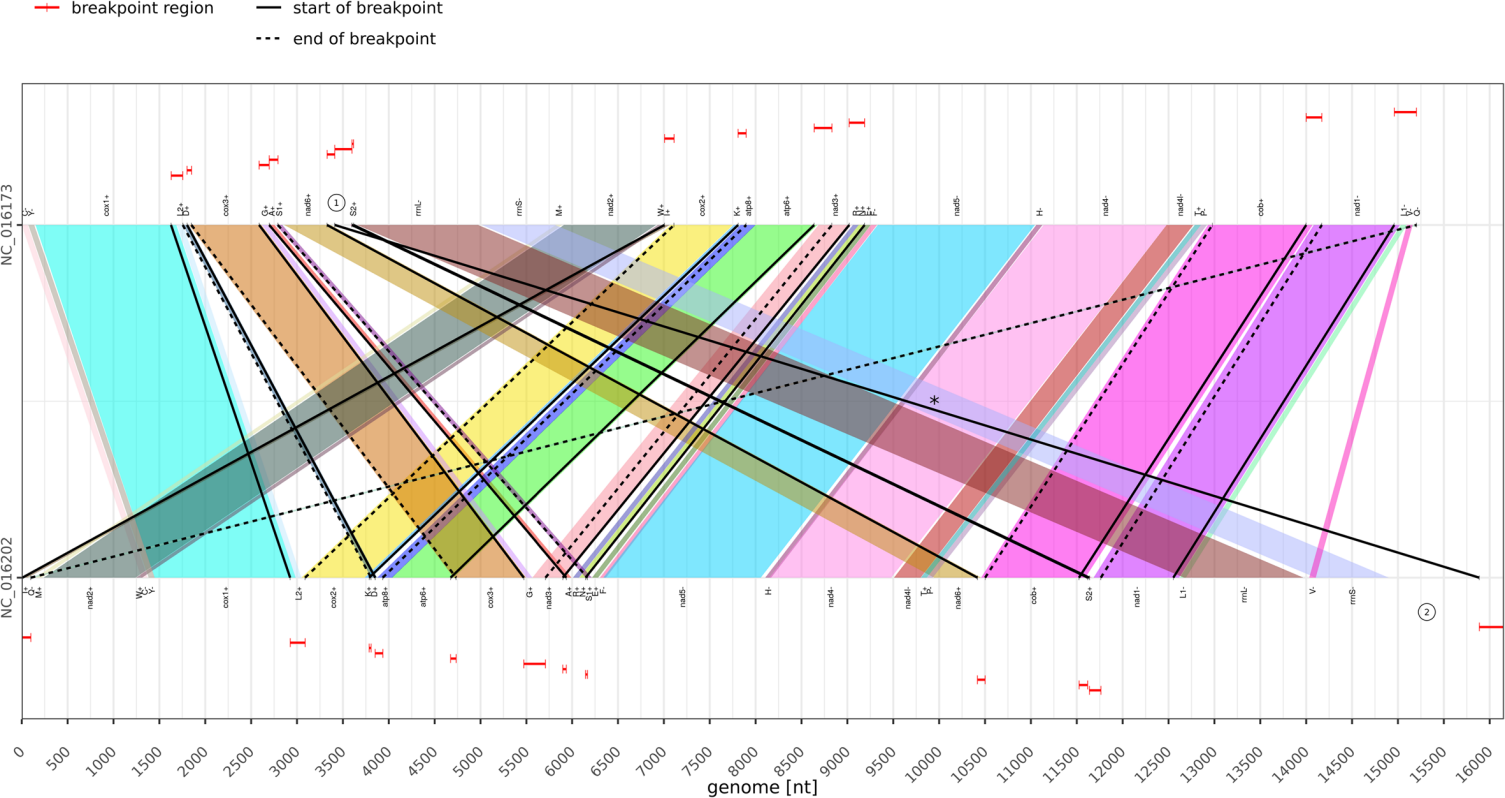
Breakpoint plot of the Nematocera data set (third case study)

In contrast to all other experiments, which used a value of $k=10$, the automatic computation routine determined a value $k=12$ for the third experiment (cf. Section  “Determination of the (*k* + 1)-mer size”). There is an overall good agreement between the DeBBI predictions and the putative breakpoint locations (cf. Figs. 11, 13). Manual inspection showed that the undetected breakpoints were missed due to high levels of sequence dissimilarity (much worse than the average $(k+1)$-mer match rate suggests) in at least one of the two genes involved. In seven of these genes, none of the $(k+1)$-mers matched between both species, even for $k=10$. Again, DeBBI identified a homolog sequence segment between two non-coding regions, which are highlighted by encircled numbers one and two in the corresponding breakpoint plot of Fig. 13. The resulting additional breakpoint region is the one that exceeds the bounds of the linear genome representation (split red interval at the beginning and end of the bottom sequence of the figure).

progressiveMauve identifies no rearrangements in the first case study. This is because it outputs a sequence of consecutive alignment blocks (see Additional file 1: Figure S15) so that all breakpoints are missed. Consequently, the corresponding EDF in Fig. 11 collapses to a single point at the origin (green triangle). In case studies 2 and 3, progressiveMauve detects a considerably smaller number of breakpoints than DeBBI (cf. Fig. 11). Long unaligned sequence segments cause the missed rearrangements for case study 2, and a mixture of long unaligned and erroneously aligned segments causes them in case study 3 (see Additional file 1: Figs. S15, S17, and S18). The incorrect alignments manifest in anti-parallel lines between the corresponding gene encoding regions in the breakpoint plot of Additional file 1: Figure S18. In contrast to the anti-parallel lines in the DeBBI breakpoint plots, which associate non-coding sequence segments to one another, these alignments cause two incorrect breakpoints.

In all three case studies progressiveMauve particularly misses breakpoints involving at least one tRNA gene, as was the case for the simulated data sets. A possible explanation could be that rearranged mitochondrial tRNAs, which are frequently also poorly conserved [23–25], would correspond to extremely short alignment blocks, which makes it very challenging to distinguish these blocks from random alignments.

#### Gene inversions

**Table 4:** Real genome gene inversion case studies

Accession IDs		lca	Genes on opposing strands	Inversion match rate (%)	Identified by DeBBI	Identified by progressiveMauve
NC_022713	NC_23799	Clupeocephala	tRNA-P	4.3	$\checkmark$	
NC_23799	NC_031827	Cyprinidae	nad6	3.1	$\checkmark$	$\checkmark$
NC_022713	NC_031827	Clupeocephala	tRNA-P	21.0	$\checkmark$	
			nad6	0.2		

Shown are the accession IDs, the lowest common ancestor (lca), the genes residing on opposing strands, the inversion match rate of these genes, and whether the corresponding IBs have been identified by DeBBI and/or progressiveMauve

The gene inversion case study was performed on three Clupeocephala mitogenomes (see Additional file 1: Figure S10 for the corresponding taxonomic tree). Each of the three species has the same gene order, but some genes are located on opposite strands (cf. Table 4). DeBBI missed one of the four inversion blocks (IBs). This was caused by the poor conservation of gene content of this block, as the corresponding inversion match rate of only $0.2\%$ indicates. In addition to this IB, progressiveMauve missed two further inversion blocks.

#### Additional experiments

We also evaluated the breakpoint predictions for the above real genome data sets using two other sequence aligner Gecko and CHROMEISTER. Both tools produced predictions of poorer quality than progressiveMauve and DeBBI in all cases. The findings are compiled in Additional file 1: Section *Evaluation with* Gecko * and* CHROMEISTER .

### Running times

All experiments were run on a desktop computer with an AMD Ryzen^™^ 7 1700 processor with 3 GHz, using two hardware threads.

#### Simulated data

**Fig. 14:**
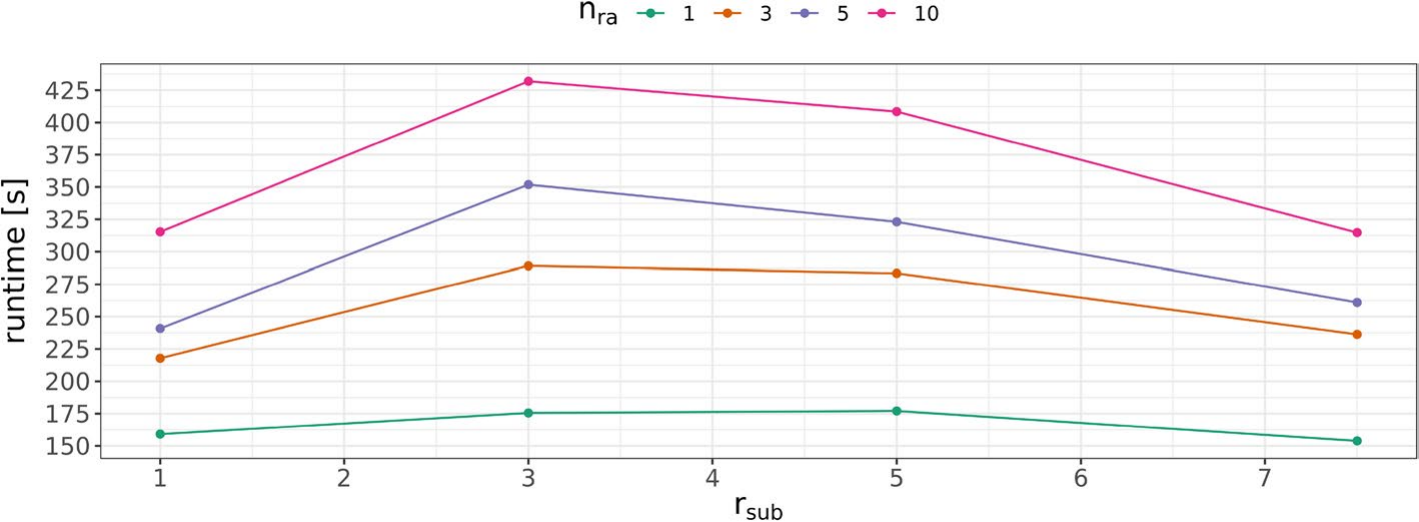
DeBBI runtimes with two threads for the synthetic gene dislocation experiments with one, three, five, and ten rearrangements $n_{\text{ra}}$ per genome and different substitution rates $r_{\text{sub}}$

Figure 14 summarizes DeBBI ’s time requirements for the dislocation experiments on the synthetic data set. The given times encompass the parsing from input fasta files, the construction of the de-Bruijn graphs for the positive strands, and all seven steps outlined in the “Methods” section to compute the breakpoint locations between the 10 input sequences per dislocation experiment.

We find that running times depend both on the level of sequence inconsistencies, as controlled by $r_{\text{sub}}$, and on the number of putative breakpoints $n_{\text{ra}}$, i.e., the discrepancy in the gene orders. A large number of putative breakpoints results in a larger number of bulge candidates that need to be analyzed so that the runtimes are longer for larger values of $n_{\text{ra}}$.

Such monotonic behavior cannot be observed for the level of sequence dissimilarities. Moderate substitution rates of $r_{\text{sub}} =3$ (i.e., the peaks of the curves) require most resources. This can be explained as follows. At low substitution rates, the de-Bruijn graph is hardly cluttered since the gene encoding regions are well-conserved, i.e., contain only few sequence dissimilarities. As a consequence, there is only a small number of BB candidates originating from random matches of unrelated sequence segments. Hence fewer candidates must be analyzed, which is reflected in shorter running times. On the other hand, while there is a lot more noise in the de-Bruijn graph for large substitution rates, which results in a larger number of initially present (mostly noisy) BB candidatesd, most of these candidates are discarded at an early stage (end of Step 4) due to insufficient alignment quality. This prevents a massive increase in runtime. Moreover, the noise also causes an increasing number of actually correct BBs to suffer from insufficient alignment quality so that these are also sorted out prematurely, hence leaving fewer breakpoint bulges for the more costly analysis after Step 4, as compared to lower substitution rates.

**Fig. 15:**
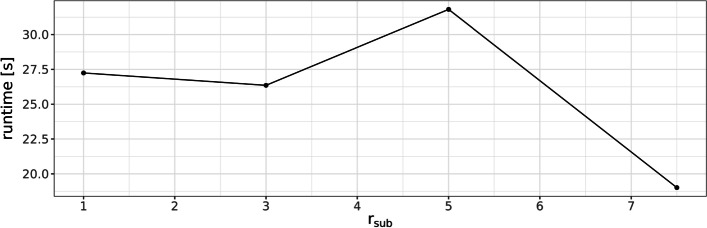
DeBBI runtimes with two threads for the synthetic gene inversion experiments with different substitution rates $r_{\text{sub}}$

The running times for detecting inversions in the synthetic data sets are summarized in Fig. 15. The time measurement includes the parsing from input fasta files, the construction of the de-Bruijn graphs of both positive and negative strands, and all steps for the computation of gene inversion breakpoints as described in the “Methods” section. Again we observe a general trend towards shorter running times for larger substitution rates. A similar argument as for the above dislocation experiments can explain this.

By comparing the above runtimes with the times required for a single hardware thread, a mean speed-up of approximately 1.45 and an efficiency of 0.73 was measured for the dislocation experiments, while a mean speed-up of approximately 1.56 and an efficiency of 0.78 was measured for the gene inversion experiments.

#### Case studies

The running times for the case studies, involving both dislocation and inversion experiments, are shown in Table 5. The measured times reflect the dependency on the number of putative breakpoints and the level of sequence inconsistency observed in the synthetic data sets.

**Table 5:** Time requirements of DeBBI with two threads for the real genome experiments

Accession IDs			Time (s)
Dislocation experiment			
NC_030373	NC_025935		40.2
NC_007894	NC_009690		98
NC_016202	NC_16173		66
Inversion experiment			
NC_022713	NC_23799	NC_031827	17.4

## Discussion

This contribution describes a new method for the detection of breakpoint locations in the nucleotide sequences of complete mitochondrial genomes. It constructs a position-annotated de-Bruijn graph of the input sequences, which is then scanned for particular bulge structures that may be associated with gene rearrangement events. Gene dislocations and gene inversions can be analyzed independently. The method is implemented in the software package DeBBI.

In-depth experiments on a comprehensive collection of simulated mitochondrial sequences demonstrate DeBBI ’s ability to identify breakpoints in species with highly, moderately, and slightly rearranged gene orders while also allowing for substantial sequence divergence. Case studies on species of different taxonomic groups further showcase DeBBI ’s applicability to real-life mitochondrial sequences. The standard multiple-sequence alignment tool progressiveMauve is used for a comparative analysis in the main manuscript. Further experimental evaluations with two additional alignment tools, CHROMEISTER and Gecko, are provided in the supplementary material (Additional file 1: Section Evaluation with Gecko and CHROMEISTER).

On both artificial and real data sets, progressiveMauve fails to discover many of the breakpoints identified with DeBBI by either creating a large alignment block that contains them or not aligning the corresponding sequence segments at all. An even smaller percentage of breakpoints is found by CHROMEISTER and Gecko in the corresponding experiments. With all three tools, in particular breakpoints involving at least one tRNA gene are often missed. This problem seems to be inherent to the identification of collinear sequence blocks, which are used to compute the breakpoint locations in these cases. In constrast, DeBBI directly locates the breaks between genes so that this issue does not occur. Since the genomics positions of mitochondrial tRNAs are rearranged much more frequently than the longer rRNA and protein-coding genes [37, 38], this is a significant advantage of DeBBI to locate gene breaks in mitogenomes.

### Future work

As the target application scenario, we focus on mitochondrial genomes where heavily rearranged gene orders are common and rearranged genes are often poorly conserved, hoping to gain insights into the underlying rearrangement mechanisms. The algorithmic idea behind the proposed approach is, however, not limited to a use on mitogenomes, but could also be applied to nuclear genomes. To optimize the result quality in such a case, the default settings for the runtime parameters might need to be appropriately adapted. This could be an interesting aspect to be explored in future studies.

The breakpoint locations identified by DeBBI could in principle also be used to create collinear blocks between the input sequences. However, if not all breakpoints are discovered, these blocks generally cannot be unambiguously generated, as outlined in Additional file 1: Section *Computing collinear blocks*. Nevertheless, creating a most consistent set of collinear blocks from a subset of the breakpoint predictions is possible (cf. Additional file 1: Section *Computing collinear blocks*). Attempting to employ the location of the remaining breakpoints to identify additional blocks is future work.

## Conclusion

DeBBI is a new tool to compute the location of gene dislocation and inversion breakpoints. It requires only nucleotide sequences as input and can be run in parallel. The core element of the underlying approach is a position-annotated de-Bruijn graph, which is searched for particular structures, called breakpoint bulges, using a novel heuristic algorithm. DeBBI produces good results for both synthetic and real-life mitochondrial sequences. By locating gene breakpoints directly rather than deducing them from alignment blocks, DeBBI often also discovers rearrangements between short, poorly conserved tRNA genes, which are frequently missed by alignment-based approaches.

We emphasize that genome alignment tools are not designed for the task of breakpoint detection. The difference in performance thus highlights the need for breakpoint detectors that can operate efficiently on divergent nucleotide sequences. While DeBBI efficiently detects correct breakpoint locations, the accuracy of breakpoint locations appears to be limited to several dozen nucleotides. If more accurate locations are required, the predictions of DeBBI could be used as input of accurate but slow alignment-based methods such as [18].

## Supplementary Information

**Additional file 1. **Section Evaluation with Gecko and CHROMEISTER.

## Data Availability

DeBBI has been released as free and open-source software under the MIT/X Consortium License. The latest source code is available at https://git.informatik.uni-leipzig.de/lfiedler/debbi-tool-for-gene-breakpoint-identification.

## Abbreviations

SV: Structural variant
MSB: Maximal synteny block
BB: Breakpoint bulge
IB: Inverted sequence blocks
