# Supplementary material for: Detecting gene breakpoints in noisy genome sequences using position-annotated colored de-Bruijn graphs
Source: BMC Bioinformatics. 2023 Jun 5;24:235. doi: 10.1186/s12859-023-05371-4 (PMC10243065; doi:10.1186/s12859-023-05371-4)
Supplement: Supplementary file 1 — Additional file 1. Section Evaluation with Gecko and CHROMEISTER. [file 12859_2023_5371_MOESM1_ESM.pdf]

Additional file 1

Lisa Fiedler, Matthias Bernt, Martin Middendorf, Peter Stadler

Additional File covering detailed information on the methods presented in the main manuscript, including alignment parameters and branch length distributions, as well as additional tables and figures to predictions analyzed and discussed in the main text.

Handling linear genomes

For linear genomes, the breakpoint detection routine differs only slightly from the approach presented in the main manuscript.

After IPs and TPs are identified in the first step of the dislocation breakpoint routine, only IP-TP combinations where the IP position annotations are smaller than the corresponding TP position annotations are used. This way, the genome bounds are not passed by either of the branches. Thereafter, the algorithm can be carried out in precisely the same manner.

For the inversion breakpoint routine, broken bulges are not considered further where $p_5 > p_6'$ or $p_5' > p_6$.

Local sequence alignments

Parameters

For the local sequence alignments, an alignment matrix with match costs of 1 and mismatch costs of -2 was used. Moreover, gap penalties of -2 for opening and extending a gap were applied. These settings are used as default settings in `BLAST` which assume 95% of sequence conservation.

To render the quality of the sequence alignments comparable, the *E-values* of the resulting alignment scores were computed. For an alignment with score S, the E-value is the expected number of alignments with score at least S,i.e., the smaller the E-value, the higher the alignment quality. The cutoff E-value should be set based on the degree of conservation of the involved species. The E-value computation involves statistical parameters λ, K, H, which for gapped alignments must be inferred from simulated sequences. These were computed by aligning a large number of random sequence tuples and recording their best scores S' and alignment lengths l'. To generate sequence tuples of representative sequence composition, long random sequences were generated by concatenating and shuffling the sequences of all Metazoan species contained in `RefSeq89` and then splitting them into sequence chunks of average genome length. The maximum-likelihood method was then applied to estimate the desired parameters by assuming an extreme value distribution of S', as suggested by [1, 2]. Alternatively, the sequences of the species involved can be used for this step. This might result in a slight accuracy gain of the statistical parameters, however, also an increase in runtime by the amount required to compute these parameters, where the computation of the alignment scores has the biggest impact as (expensive) sequence alignments need to be conducted. For all of the experiments conducted in this study, using the sequences of the involved species had no impact on the produced results. This is thus only advisable for atypical genomes. The parameters can also be supplied manually.

Banded alignments constrain the search space to a short band around the diagonal of the scoring matrix. A bandwidth of 40 nt proved to produce good results in practice while allowing to greatly reduce the computation time, which is why this is used as default value. Indeed, conducting a complete sequence alignment instead produced the same predictions in all experiments performed in this study.

Aligning the flanking sequence blocks in step 4

To explain how the sequence segments are determined for the sequence alignment at the bulge flanks (step 4), consider BB B_1 of Figure 2 in the main manuscript. For this case, the alignment is triggered between r_2 from $\mathrm{pred}(5, \rho)$ to $\mathrm{pred}(5, 1) = 4$ and r_1 from $\mathrm{pred}(5, \rho)$ to $\mathrm{pred}(5, 1) = 4$ for the left segment and between r_2 from $\mathrm{pred}(15, k)$ to $\mathrm{pred}(15, k + \rho)$ and r_1 from $\mathrm{pred}(7, k)$ to $\mathrm{pred}(7, \rho)$ for the right segment. The shift by k is because the position annotated on an edge refers to the last nucleotide of the $(k + 1)$-mer, not the entire subsequence.

The flanking MSB s must consist of at least one gene of size $|\,\mathrm{g_{min}}\,|$. We hence use flanking sequence blocks of the order of this length, in detail $\rho = |\,\mathrm{g_{min}}\,| + 20$. The rational of using a slightly longer block is to account for the fact that the match quality generally decreases in the vicinity of the gene boundaries and this region of low match quality tends to be wider for longer genes, such that long genes might score too poorly to be identified. 20 additional nucleotides proved to produce good results, while still keeping the sequences for the alignment at a moderate size and thus keeping the required runtimes for the alignments at bay.

The alignment is enforced to end at $\mathrm{pred}(5)$ in r_2 and $\mathrm{pred}(5)$ in r_1 for the left alignment and start at $\mathrm{pred}(15, k)$ in r_2 and $\mathrm{pred}(7, k)$ in r_1 for the right alignment, since the MSB s should directly adjoin the BB. Note that if ρ exceeds the collinear region at the flanks to a small extent, sequence segments that go beyond this region will generally not be included in the resulting local alignment and in particular the match quality is not degraded, as opposed to a global alignment.

Branch lengths

The precise length of the single-color branch depends on the degree of conservation between the genomes under consideration, the presence of intergenic regions, and whether or not the genes involved in the BB and the two entangled BBs start or end with the same encoding subsequence. If none of the above apply, the single-color branc is of length k and consists of transition $(k+1)$-mer s where the prefixes correspond to the last few nucleotides of the first breakpoint gene and suffixes correspond to the first few nucleotides of the second breakpoint gene. Figure S1 shows that some of such cases also occurred in the experiments of the synthetic dataset. It can be observed that for low and moderate substitution rates most of the BBs have branches of length $|b_s| = k = 10$. High substitution rates result in a broadening of the peak towards longer branch lengths. None of the branches in the case studies had lengths smaller than k. The corresponding branch length distribution is shown in Figure S2.

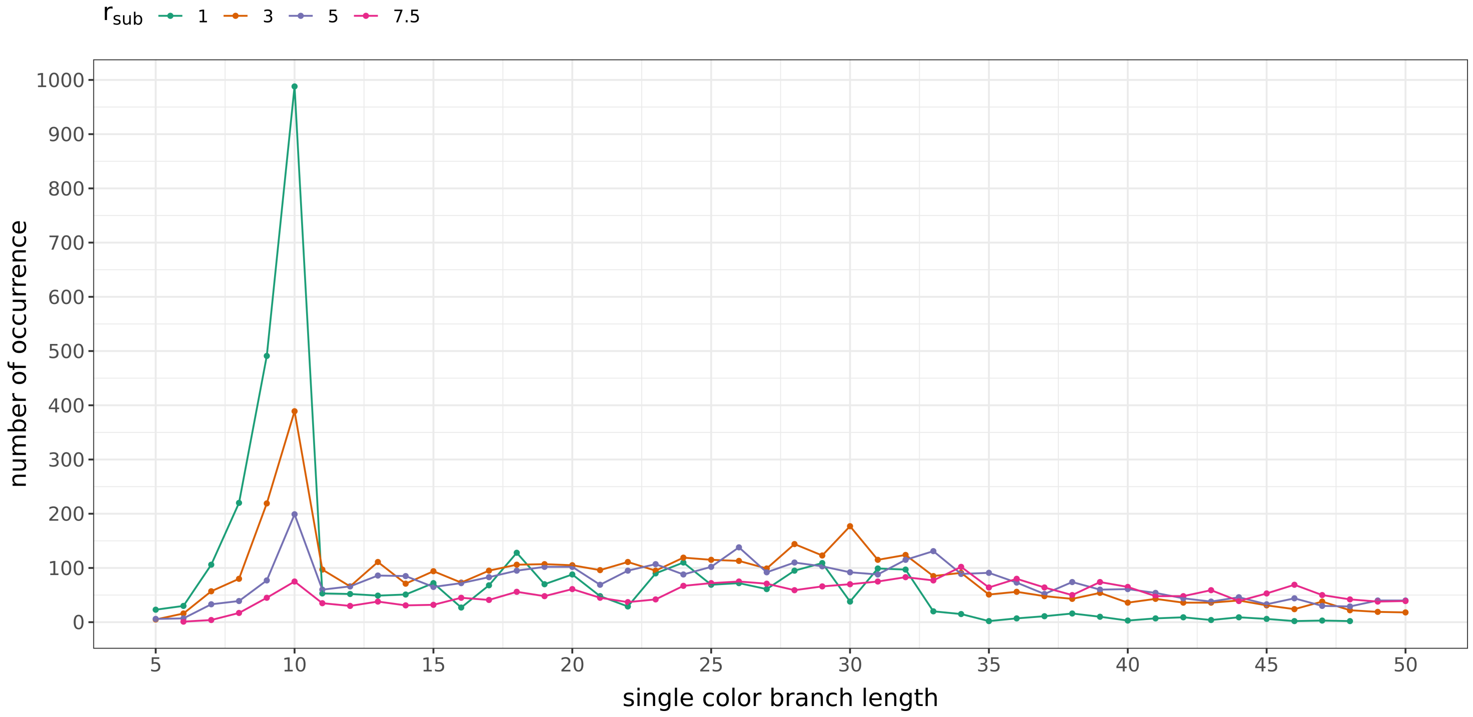

Figure S1: Branch length distribution for the synthetic datasets. Shown are only branch lengths up to size 50.

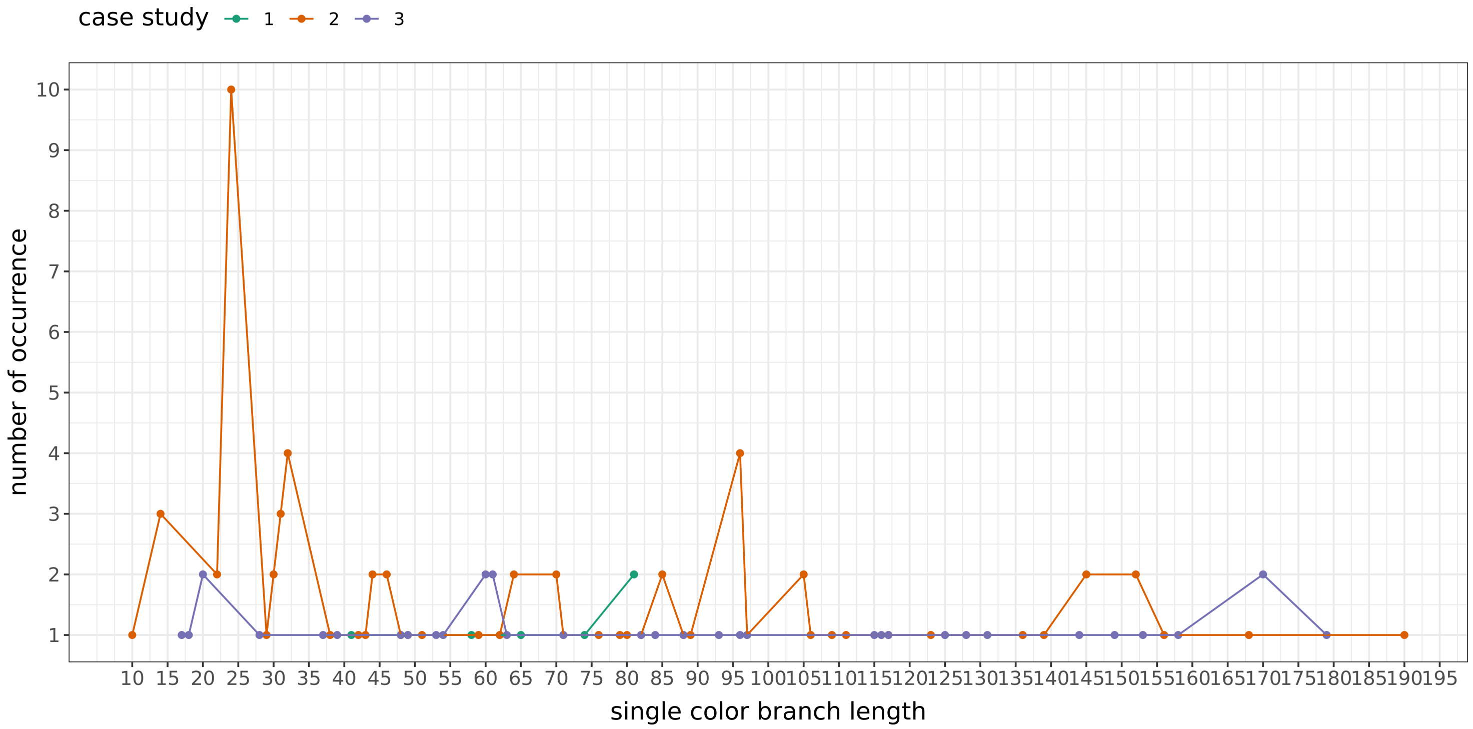

Figure S2: Branch length distribution for the dislocation case studies.

Algorithms of step 5

Like in step 4, only the first ρ vertices of the first and the last ρ vertices of the second putative two-color paths are considered. We call the corresponding r_2 subpaths P_{a}^{3} and P_{a}^{4}, respectively.

Alignment anchor detection

Input: An r_2 reference path $\mathrm{P}_{\mathrm{ref}}$, a genome r_1
Output: A set $\mathcal{P}$ of 2-color paths of r_1 and r_2

1 $E \leftarrow$ list of all edges (v, v', r_2, p) on $\mathrm{P}_{\mathrm{ref}}$
2 sort E in ascending order with respect to p
3 **foreach** $e = (v, v', r_2, p)$ **in** E **do**
4     $M \leftarrow$ retrieve set of matching edges (v, v', r_1, p')
5   **if** $M = \emptyset$ **then**
6   remove e from E
7   **continue**
8     $\mathcal{L}_1^e \leftarrow$ set of positions in M in ascending order
9     $\mathcal{L}_2^e \leftarrow \{\mathrm{succ}(p', 1)$ for each p' in $\mathcal{L}_1^e\}$
10 `// Mark edges in` E `with collinear successor`
11 **for** $i \leftarrow 1$ **to** $|E| - 1$ **do**
12   **if** $\mathcal{L}_2^{E[i]} = \mathcal{L}_1^{E[i+1]}$ **then** mark $E[i]$
13 $R \leftarrow$ empty list
14 `// Remove unmarked edges following a block of marked edges`
15 **for** $i \leftarrow 2$ **to** $|E|$ **do**
16   **if not** `marked(`$E[i]$`)` **and** `marked(`$E[i-1]$`)` **then** append $E[i]$ to R
17 $E \leftarrow E$ with entries in R removed
18 `// Compute group identifier`
19 **for** $i \leftarrow 1$ **to** $|E|$ **do**
20     $d[i] \leftarrow p - i$
21     $d'[i] \leftarrow \mathcal{L}_2^{E[i]}[1] - i$
22 `// Group entries in` E `with equal values of` (d, d') `retaining their order`
23 $\mathcal{G} \leftarrow \emptyset$
24 $G \leftarrow$ empty list
25 **for** $i \leftarrow 1$ **to** $|E|$ **do**
26   append $(E[i], \mathcal{L}_1^{E[i]}, \mathcal{L}_2^{E[i]})$ to G
27   **if** $d[i{+}1] \neq d[i]$ **or** $d'[i{+}1] \neq d'[i]$ **then** `// start new group`
28         $\mathcal{G} \leftarrow \mathcal{G} \cup \{G\}$
29         $G \leftarrow$ empty list
30 $\mathcal{P} \leftarrow \emptyset$
31 **foreach** G **in** $\mathcal{G}$ **do**
32   **if** `marked(`$G[1]$`)` **then** `// all elements of` G `are marked`
33   `// In this case` $\mathcal{L}_1$ `is of same size for all elements in` G
34   **for** $i \leftarrow 1$ **to** $|$`getL1(`$G[1]$`)`$|$ **do**
35             $P_{r_2} \leftarrow (r_2,$ `getP(`$G[1]$`)`, succ(`getP(`$G[-1]$`)`$, 1))$
36             $P_{r_1} \leftarrow (r_1,$ `getL1(`$G[1]$`)`$[i]$, `getL2(`$G[-1]$`)`$[i])$
37             $\mathcal{P} \leftarrow \mathcal{P} \cup \{P_{r_2}, P_{r_1}\}$ `// add 2-color path to result set`
38   **else** `// no element of` G `is marked`
39   **foreach** g **in** G **do**
40   **for** $i \leftarrow 1$ **to** $|$`getL1(`g`)`$|$ **do**
41                 $P_{r_2} \leftarrow (r_2,$ `getP(`g`)`, `getP(`g`)`$)$
42                 $P_{r_1} \leftarrow (r_1,$ `getL1(`g`)`$[i]$, `getL1(`g`)`$[i])$
43                 $\mathcal{P} \leftarrow \mathcal{P} \cup \{P_{r_2}, P_{r_1}\}$ `// add 2-color path to result set`
44 **return** $\mathcal{P}$

Algorithm 1: Identifying 2-color paths

To determine whether the corresponding r_1 subpaths exist and, if so, whether homologous regions are present, we use Algorithm 1. The procedure is applied independently to the paths P_{a}^{3} and P_{a}^{4}, unless b_{a} is shorter than $|P_{\mathrm{a}}^{1}| + |P_{\mathrm{a}}^{2}| + 2\rho$, i.e., if the two paths overlap. This is the case if (different from the situation in BB B_1) there is only a single MSB on b_{a}. Then, both paths are combined into a single path $P_{\mathrm{a}}^{3,4}$, which contains the overlapping edges only once, and Algorithm 1 is applied to the combined path $P_{\mathrm{a}}^{3,4}$.

The general idea is to create alignment anchors on the r_2 *reference paths* $\mathrm{P}_{\mathrm{ref}} \in \{P_{\mathrm{a}}^{3}, P_{\mathrm{a}}^{4}, P_{\mathrm{a}}^{3,4}\}$ by combining consecutive $(k+1)$-mer matches of r_1 using a local sorting strategy. Given an input reference path $\mathrm{P}_{\mathrm{ref}}$, the algorithm first retrieves all matching $(k+1)$-mers of r_1 along this path (line 4) and discards all of r_2's edges without matches (line 6). For each of the remaining edges e of r_2, which are stored in ascending order in a list E, an ordered set $\mathcal{L}_1^e$ of the positions of matching $(k+1)$-mers is generated (line 8), along with a second set $\mathcal{L}_2$ consisting of the succeeding positions stored in $\mathcal{L}_1^e$ (line 9). For instance, in the example scenario depicted in fig. S3, two r_1 edges with positions 44 and 249 match at r_2's edge $E[1]$, resulting in sets $\mathcal{L}_1^e = \{44, 249\}$ and $\mathcal{L}_2^e = \{45, 250\}$. If $\mathcal{L}_1^e = \mathcal{L}_2^{e'}$ for two consecutive edges e and e' in E, the edges are collinear with the sequences of matching r_1 edges at e and e'. This is the case for above

index in E	1	2	3	4	5	6	7
position p in r_2	3	4	5	6	8	9	10
$\mathcal{L}_1$	$\{44, 249\}$	$\{45, 250\}$	$\{46, 251\}$	$\{60\}$	$\{61\}$	$\{90, 95\}$	$\{91\}$
$\mathcal{L}_2$	$\{45, 250\}$	$\{46, 251\}$	$\{47, 252\}$	$\{61\}$	$\{62\}$	$\{91, 96\}$	$\{92\}$
marked	✓	✓					
keep	✓	✓		✓	✓	✓	✓
d	2	2	-	2	3	3	3
d'	43	43	-	55	56	84	84
group	a	a	-	b	c	d	d
type	m	m	-	u	u	u	u
2-color paths	$\mathbf{\{(r_2, 3, 5), (r_1, 44, 47)\}}$<br>$\mathbf{\{(r_2, 3, 5), (r_1, 249, 252)\}}$		-	$\{(r_2, 6, 6), (r_1, 60, 60)\}$	$\{(r_2, 8, 8), (r_1, 61, 61)\}$	$\{(r_2, 9, 9), (r_1, 90, 90)\}$<br>$\{(r_2, 9, 9), (r_1, 95, 95)\}$	$\{(r_2, 10, 10), (r_1, 91, 91)\}$
chains	-		-	$\mathbf{(\{(r_2, 6, 6), (r_1, 60, 60)\}, \{(r_2, 8, 8), (r_1, 61, 61)\})}$		$(\{(r_2, 9, 9), (r_1, 90, 90)\}, \{(r_2, 10, 10), (r_1, 91, 91)\})$	

Figure S3: Identification of 2-color paths for an input single color path $(r_2, 1, 10)$ using Algorithm 1 and generation of alignment candidates using Algorithm 2. Edges of r_2 without matching $(k+1)$-mers of r_1, comprising r_2's edges with $p \in \{1, 2, 7\}$, have already been removed in list E. Consecutive edges of r_2 that are assigned to the same group are labeled with the same identifier a,b,c, or d. The type specifies whether the group consists only of marked (m) or unmarked (u) edges. The resulting 2-color paths which are output by Algorithm 1 are listed below the solid line. Given these 2-color paths, two chains are generated by Algorithm 2. The longest chain and the two longest paths are highlighted in red bold font. Since they all have the same length of three, alignment candidates are computed for each of them. Alignments of r_2's subsequence form 1 to 10 are thus conducted with r_1's subsequence from 42 to 52, with r_1's subsequence from 247 to 257, and with r_1's subsequence from 55 to 64.

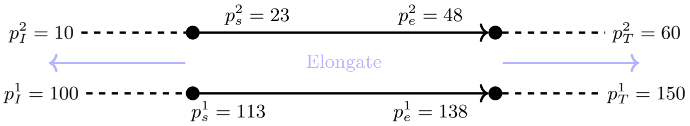

Figure S4: Linear extension of an anchor point $\{(r_2, 23, 48), (r_1, 113, 138)\}$ for an input reference path $(r_2, 10, 60)$. The resulting position array of r_1 that would be output by Algorithm 2 is $[100, 150]$. In the subsequent alignment, r_2's subsequence from position 10 to 60 would be aligned with r_1's subsequence from position 100 to 150.

edge $E[1]$. Edges $E[1]$ and $E[2]$ are thus collinear to two sequences of matching edges, the first sequence comprised of two r_1 edges with positions 44 and 45 and the second one of two r_1 edges with positions 249 and 250. The algorithm marks such edges $E[i]$ with equal sets $\mathcal{L}_1$ and $\mathcal{L}_2$ (line 12). Blocks of consecutive marked edges are part of the same collinear region. These are thus grouped together (lines 23-29), and a two-color path for each collinear sequence of matching $(k+1)$-mers is output by the method (lines 32-37). Above edges $E[1]$ and $E[2]$ are part of such a collinear region, yielding 2-color paths $\{(r_2, 3, 5), (r_1, 44, 47)\}$ and $\{(r_2, 3, 5), (r_1, 249, 252)\}$. Consecutive unmarked edges indicate different independent collinear regions so that the algorithm outputs individual two-color paths for each of them. All remaining two-color paths of fig. S3 are created this way.

Input: A set $\mathcal{P}$ of 2-color paths $P = \{(r_2, p_s^2, p_e^2), (r_1, p_s^1, p_e^1)\}$ for a reference path (r_2, p_I^2, p_T^2)
Output: A set of alignment candidates $\mathcal{A}$

1 $\mathcal{L}_{\text{adj}} \leftarrow \emptyset$
2 **foreach** P **in** $\mathcal{P}$ **do**
3   **foreach** $\hat{P}$ **in** $\mathcal{P}$ **do**
4   **if** $\texttt{distance}(\texttt{p}_\texttt{e}^2(P), \texttt{p}_\texttt{s}^2(\hat{P})) \leq k+1$ **and** $\texttt{distance}(\texttt{p}_\texttt{e}^1(P), \texttt{p}_\texttt{s}^1(\hat{P})) \leq k+1$ **then**
5       $\mathcal{L}_{\text{adj}} \leftarrow \mathcal{L}_{\text{adj}} \cup \{(P, \hat{P})\}$ // add to adjacency list
6 $\mathcal{S} \leftarrow \{(P, \hat{P}) \in \mathcal{L}_{\text{adj}} \mid \nexists (P', P) \in \mathcal{L}_{\text{adj}}\}$ // no predecessor exists
7 $\mathcal{L}_{\text{adj}} \leftarrow \mathcal{L}_{\text{adj}} \setminus \mathcal{S}$
8 **do**
9   $\mathcal{S}' \leftarrow \mathcal{S}$
10   // remove elements that have been visited before
11   $\mathcal{L}_{\text{adj}} \leftarrow \{(P', \hat{P}) \in \mathcal{L}_{\text{adj}} \mid \nexists (P, \hat{P}) \in \mathcal{S}\}$
12   // create chains of 2-color paths
13   **foreach** $(P, \hat{P})$ **in** $\mathcal{S}$ **do**
14   **foreach** (P', P'') **in** $\mathcal{L}_{\text{adj}}$ **with** $P' = \hat{P}$ **do**
15       $\mathcal{S} \leftarrow (\mathcal{S} \setminus \{(P, \hat{P})\}) \cup \{(P, P'')\}$
16 **while** $\mathcal{S}' \neq \mathcal{S}$
17 $\mathcal{C} \leftarrow \text{argmax}_{(P,\hat{P}) \in \mathcal{S}}\, \texttt{distance}(\texttt{p}_\texttt{s}^2(P), \texttt{p}_\texttt{e}^2(\hat{P}))$ // select longest chains
18 $\mathcal{T} \leftarrow \text{argmax}_{P \in \mathcal{P}}\, \texttt{distance}(\texttt{p}_\texttt{s}^2(P), \texttt{p}_\texttt{e}^2(P))$ // select longest input 2-color paths
19 $d_\mathcal{C} \leftarrow$ length of chains in $\mathcal{C}$
20 $d_\mathcal{T} \leftarrow$ length of paths in $\mathcal{T}$
21 $\mathcal{AP} \leftarrow \emptyset$
22 // Select longest chains or input 2-color paths as anchor points $\mathcal{AP}$
23 **if** $d_\mathcal{C} < d_\mathcal{T}$ **then** $\mathcal{AP} \leftarrow \mathcal{T}$
24 **else if** $d_\mathcal{C} > d_\mathcal{T}$ **then** $\mathcal{AP} \leftarrow \{P \mid (P, \hat{P}) \in \mathcal{C}\}$
25 **else** $\mathcal{AP} \leftarrow \mathcal{T} \cap \{P \mid (P, \hat{P}) \in \mathcal{C}\}$
26 $\mathcal{A} \leftarrow \emptyset$
27 **foreach** $P = \{(r_2, p_s^2, p_e^2), (r_1, p_s^1, p_e^1)\}$ **in** $\mathcal{AP}$ **do**
28   $p_I^1 \leftarrow \text{pred}(p_s^1, \texttt{distance}(p_I^2, p_s^2))$
29   $\mathcal{A} \leftarrow \mathcal{A} \cap [p_I^1, p_I^1 + \texttt{distance}(p_I^2, p_T^2)]$
30 **return** $\mathcal{A}$

Algorithm 2: Combine 2-color paths

Alignment anchor chaining

A point mutation distinguishing r_1 and r_2 in general results in $k+1$ unmatched edges in the de-Bruijn graph (cf. Figure 3 of the main manuscript). As a consequence, there may be multiple separate 2-color paths that are output by Algorithm 1, which are actually part of the same homologous region. Considered on their own, these individual paths may, because of too short path lengths, not be sufficient indicators for homology.

Thus, Algorithm 2 creates chains of two-color paths that cover as much of the reference path $\mathrm{P}_{\text{ref}} =: (r_2, p_I^2, p_T^2)$ as possible. Initially, all 2-color paths are paired that are separated by a distance of at most $k+1$, creating an adjacency list (lines 2-5). These pairs are chained as long as possible, only storing the first and last pair of the chain (lines 13-15). This is the first time a path traversal is required. However, given that paths of $(k+1)$-mers rather than single $(k+1)$-mers are used to form the chain, and the region to be covered has a maximum length of ρ, this traversal requires only very few iterations. In fact, in the course of the study, fewer than ten iterations were needed in most cases.

The longer the chain, the larger the portion of the input path that is covered by the chain, and the more likely it is that the chain corresponds to a homologous region. Hence, only the longest chains are selected as anchor points (lines 17 and 24). Some of the input 2-color paths may not have been included in the adjacency list as there is no other sufficiently close 2-color path. However, these paths

might on their own be longer than the longest of the identified chains. In such a case, the longest of these unchained paths are used as anchor points instead (line 23). If the longest unchained paths and the longest chains are of the same length, both are used as anchor points (line 25). This is the case in the example of fig. S3. The affected paths and chains are highlighted in bold red font. Lastly, the anchor points are linearly extended to cover the input reference path (r_2, p_I^2, p_T^2) completely (lines 27-29). An example is illustrated in fig. S4.

The position ranges of r_1 resulting from such an extension are the final output of the algorithm. They determine the locations of the subsequences of r_1, which are used in a subsequent banded local sequence alignment with r_2's subsequence from p_I^2 to p_T^2 (for an example confer fig. S3). Duplicate subsequences of r_1, which may be present due to repeats, are aligned only once to save computation time. The considered BB candidate is discarded if there are only low-quality alignments. Otherwise, all of its good-quality alignments are regarded as homologous regions.

Shifted breakpoint bulge candidates

At the end of step 6, there may still be *shifted* BB candidates contained in the set of remaining candidates. Consider Figure S5: Sequence inconsistencies within gene g_2 result in an inconsistency bulge (cf. Figure 3 of the main manuscript) between vertices v_c and v_d. This may result in two candidates for breakpoint $(g_1, g_2)_{2,1}$. One of them is the correct BB B between vertices v_a and v_b. The second one is a spurious candidate B' between vertices v_a and v_d. Such an incorrect candidate emerges if the following two conditions are met. First, the alignment quality on the right flank of B' (step 4) is sufficiently high because the inconsistency region is close enough to the start of g_2 so that only a small portion of g_2 is missing in the alignment. And second, the distance between v_f and v_b is small enough for reference path P_a^4 of step 5 to start somewhere in between v_e and v_f and terminate at v_c so that the corresponding local sequence alignment would encompass the small region between v_f and v_b with gaps or mismatches, but still yield a decent alignment score. Usually, B' would be discarded in such a case in step 6, due to the overlap of its "branches" b_s and b_a between v_b and v_c. However, since this region between v_b and v_c is very small, the corresponding alignment generated at the end of step 6 may not be sufficient to detect this region as homologous.

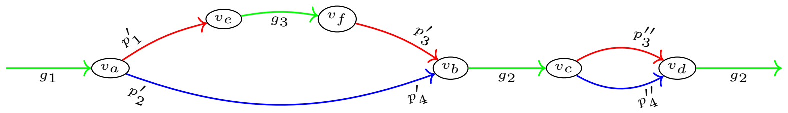

Figure S5: Formation of a shifted bulge caused by an inconsistency bulge close to the end of gene g_2. In addition to the correct candidate between v_a and v_b, a second spurious candidate between v_a and v_d exists for the BB of breakpoint $(g_1, g_2)_{2,1}$ at the end of step 6.

Impacts of the $(k+1)$-mer size on the runtime and result accuracy

Generally, an approximate exponential growth of the runtimes can be expected for decreasing values of k. This trend can also be observed for the three conducted dislocation breakpoint experiments, as shown by Figure S6, which shows the required scaled runtimes for decreasing values of k. The smallest k value per experiment was chosen so that runtimes of one hour were not exceeded.

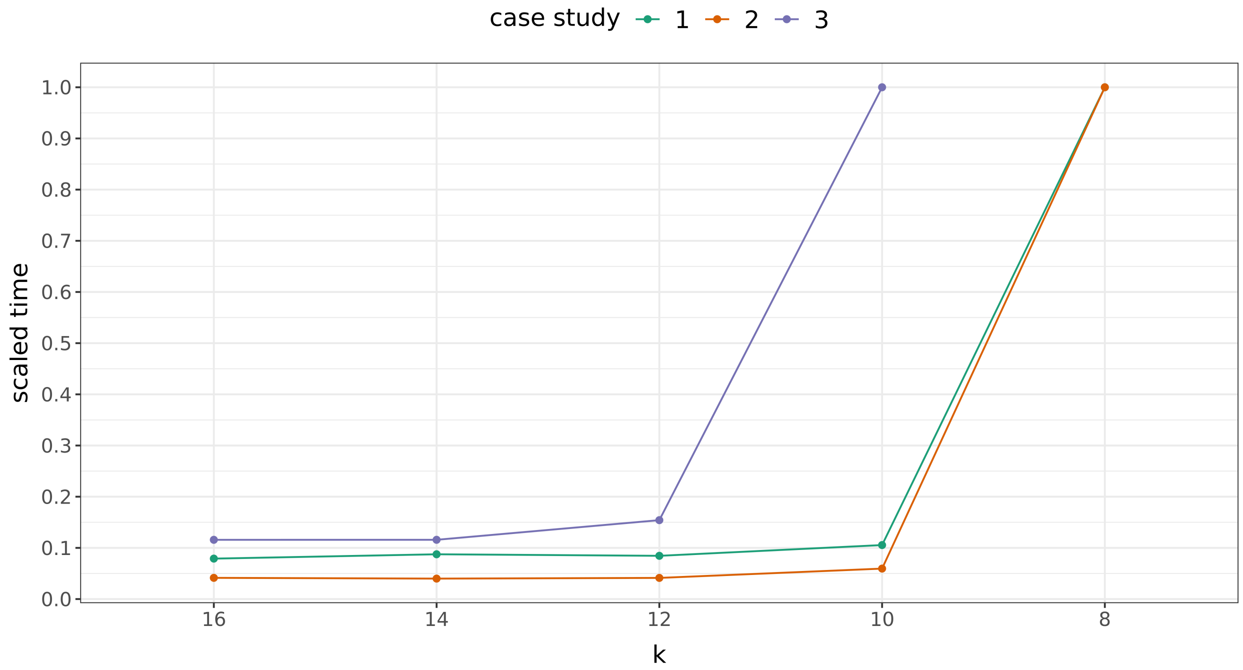

Figure S6: Scaled running times of the dislocation breakpoint case studies for decreasing values of k. The scaled runtimes are given as the required runtime for a set of genomes (case study 1, 2, or 3) and value of k divided by the longest runtime for this set of genomes among all values of k.

A smaller value of k does not necessarily improve the accuracy of the results. This is because lowering the $(k+1)$-mer size also increases the number of random matches between unrelated sequence segments. Thus a compromise between a too-large value, concealing many sequence similarities among the genomes, and a too-small value, cluttering the graph with many random matches must be found. To automate the process of deciding on a suitable value for k, an automatic computation routine can determine this value at the start of `DeBBI`. Figure S7 summarizes the result quality for the different k values by means of empirical distribution functions of distances of identified to putative breakpoints. It demonstrates that in most cases, the automatic routine selected k so that the largest number of breakpoints could be identified. These choices also avoided long runtimes, as can be seen in Figure S6.

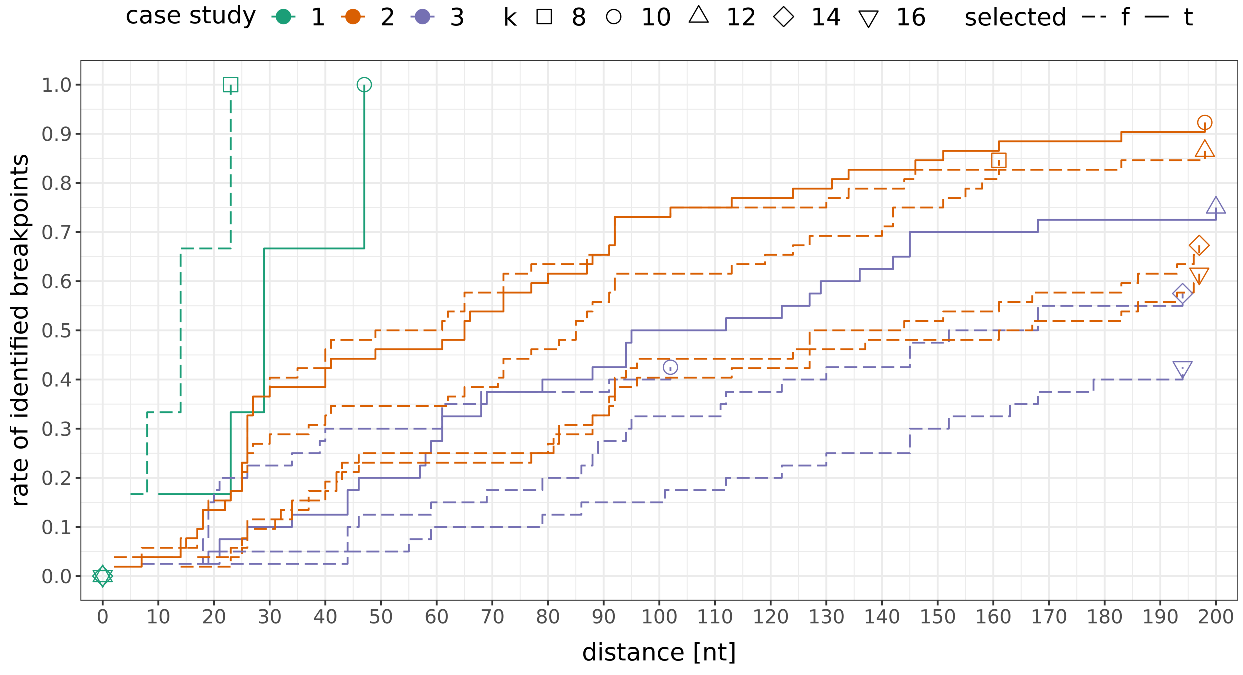

Figure S7: Empirical distribution functions (EDFs) of distances of identified to putative breakpoints, normalized to the total number of putative breakpoints for the dislocation breakpoint case studies and various values of k. The EDFs for the k values that were selected by the automatic computation routine are displayed as solid lines.

Computing collinear blocks

The breakpoint locations can in principle also be used to compute collinear blocks. In detail, the sequence segment between two successive breakpoints in one sequence forms a collinear block with the corresponding sequence segment in the other sequence. For instance, consider the two consecutive breakpoints $(g_2, g_3)_{1,2}$ and $(g_4, g_5)_{1,2}$ shown in Figure S8. In this case, the corresponding collinear block is formed by the sequence segment from positions 20 to 39 in r_1 and the sequence segment from positions 10 to 29 in r_2, corresponding to the two consecutive genes g_3 and g_4. If, as is the case in this example, all breakpoints have been identified, all collinear blocks can be constructed in this way. In this example this would result in three collinear blocks.

However, in the course of the method execution, some of the actually correct breakpoints $(g_i, g_j)_{1,2}$ might get discarded because one of the flanking genes g_i or g_j is not sufficiently conserved to be distinguished from random sequence matches. However, of the two entangledbreakpoints, breakpoint $(g_i, g_m)_{2,1}$ could still be retained if only g_j is not sufficiently conserved and breakpoint $(g_n, g_j)_{2,1}$ could still be retained if only g_i is not sufficiently conserved. Consequently, there will be some inconsistencies, in the form of overlaps, between the collinear blocks computed as described above. A most consistent set of collinear blocks can be derived by computing all collinear blocks from the breakpoints of r_1 with respect to r_2 and computing all collinear blocks from the breakpoints of r_2 with respect to r_1 and outputting only the blocks that are contained both times.

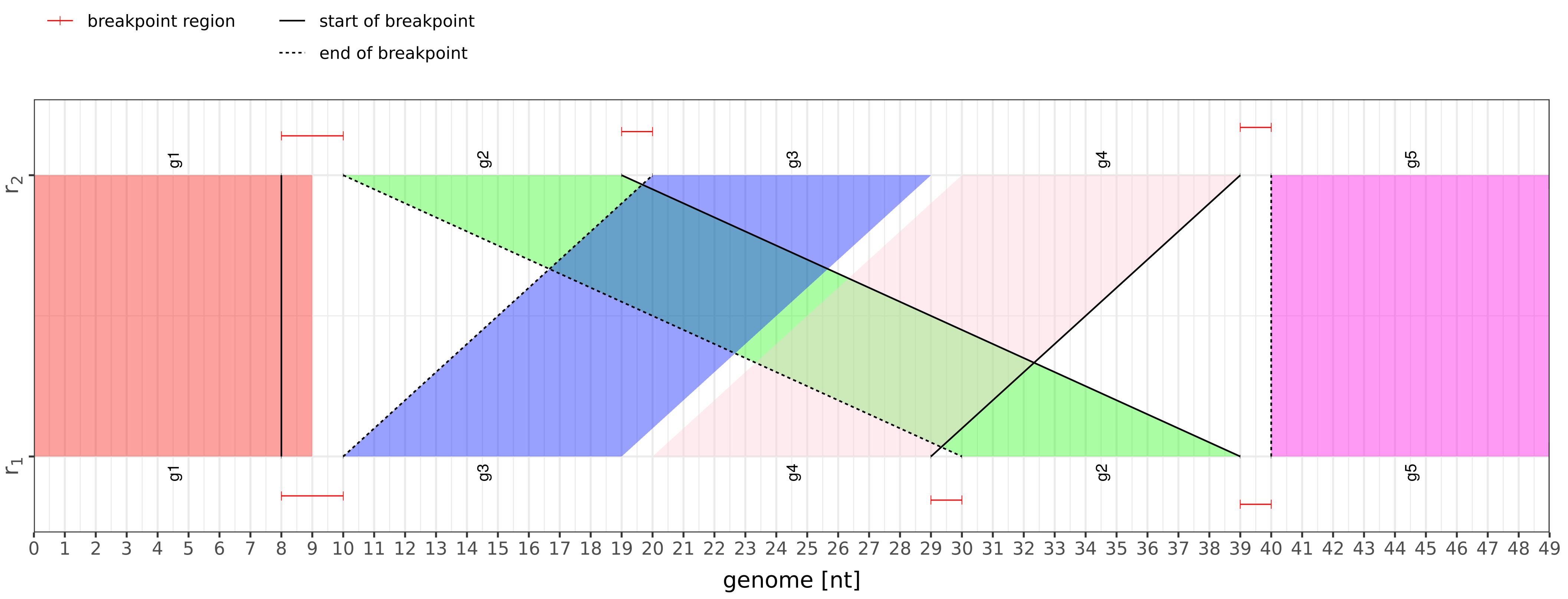

Figure S8: Example breakpoint plot.

Taxonomic Trees

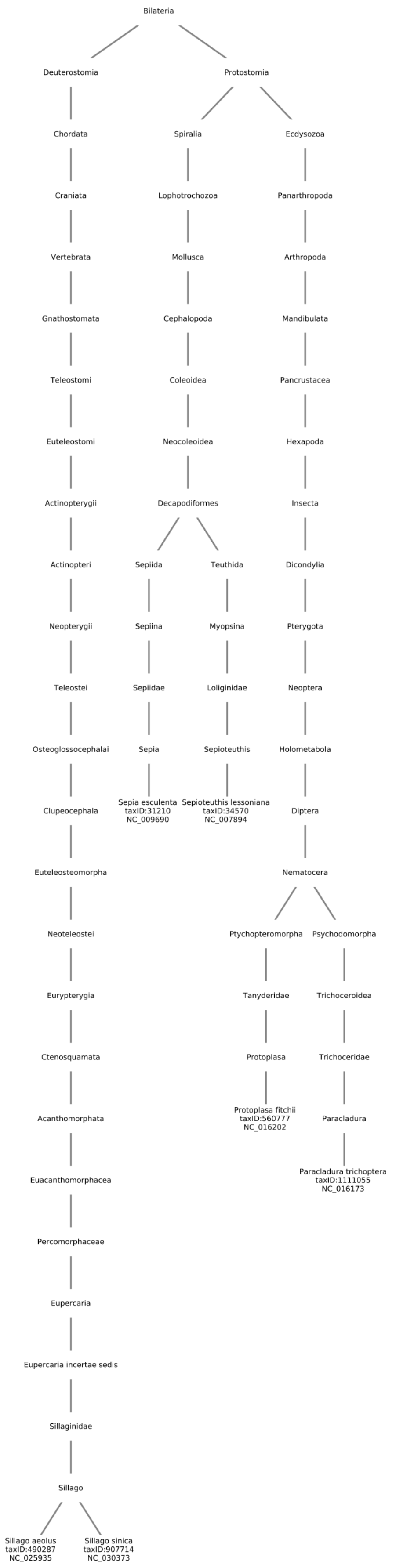

Figure S9: Taxonomic tree of species of the gene dislocation experiments.

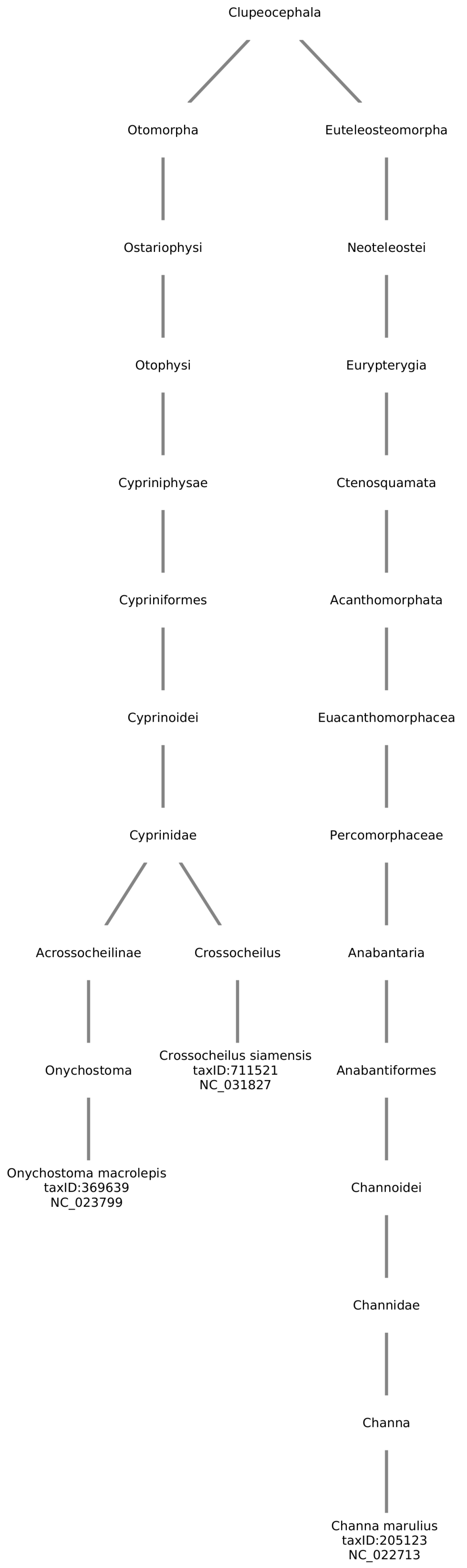

Figure S10: Taxonomic tree of species of the gene inversion experiment.

Syntethic datasets

Table S1 summarizes the mean $(k+1)$-mer match rates and $(k+1)$-mer inversion match rates that were measured for all rates of substitution r_{sub} of the synthetic data set. The table does not display $(k+1)$-mer match rates for the different gene orders since they coincide for the same value of r_{sub} when rounded to two decimal places. This is because gene rearrangements only affect the few transition $(k+1)$-mers involved in a breakpoint so that the number of introduced gene dislocations hardly impacts the $(k+1)$-mer match rates.

Table S1: Average $(k+1)$-mer match rates and $(k+1)$-mer inversion match rates for the different substitution rates r_{sub}. This serves as means to compare the level of sequence similarity of the simulated data sets with the level of sequence similarity of real genomes.

r_{sub}	1	3	5	7.5
mean pairwise match rate	0.79	0.52	0.34	0.15
mean inversion match rate [%]	71.0	44.0	23.0	2.0

Gene orders

Gene orders of dislocation case study 1 (Sillago)

NC_030373
F rrnS V rrnL L2 nad1 I -Q M nad2 W -N -A -C -Y cox1 -S2 D cox2 K atp8 atp6 cox3 G nad3 R nad4l nad4 H S1 L1 nad5 -nad6 -E cob T -P
NC_025935
F rrnS V rrnL L2 nad1 I -Q M nad2 W -A -N -C -Y cox1 -S2 D cox2 K atp8 atp6 cox3 G nad3 R nad4l nad4 H S1 L1 nad5 -nad6 -E cob T -P

Gene orders of dislocation case study 2 (Decapodiformes)

NC_007894
cox3 nad3 -S2 -cob -nad6 -P -nad1 -Q K S1 nad2 cox1 -C -Y -E N cox2 -M R -F -nad5 -nad4 -nad4l T -L2 -G I -rrnL -V -rrnS -W A D atp8 atp6 -H -L1
NC_009690
cox3 K A R S1 nad2 cox1 cox2 atp8 atp6 -F -nad1 -L2 -L1 -rrnL -V -rrnS -C -Y -Q -G N I nad3 D -nad5 -H -nad4 -nad4l T -S2 -cob -nad6 -P -M -W -E

Gene orders of dislocation case study 3 (Nematocera)

NC_016173
-C -Y cox1 L2 D cox3 G A S1 nad6 S2 -rrnL -rrnS M nad2 W I cox2 K atp8 atp6 nad3 R N E -F -nad5 -H -nad4 -nad4l T -P cob -nad1 -L1 -V -Q
NC_016202
I -Q M nad2 W -C -Y cox1 L2 cox2 K D atp8 atp6 cox3 G nad3 A R N S1 E -F -nad5 -H -nad4 -nad4l T -P nad6 cob S2 -nad1 -L1 -rrnL -V -rrnS

Gene orders of inversion case study

NC_022713
F rrnS V rrnL L2 nad1 I -Q M nad2 W -A -N -C -Y cox1 -S2 D cox2 K atp8 atp6 cox3 G nad3 R nad4l nad4 H S1 L1 nad5 -nad6 -E cob T P
NC_023799
F rrnS V rrnL L2 nad1 I -Q M nad2 W -A -N -C -Y cox1 -S2 D cox2 K atp8 atp6 cox3 G nad3 R nad4l nad4 H S1 L1 nad5 -nad6 -E cob T -P
NC_023799
F rrnS V rrnL L2 nad1 I -Q M nad2 W -A -N -C -Y cox1 -S2 D cox2 K atp8 atp6 cox3 G nad3 R nad4l nad4 H S1 L1 nad5 nad6 -E cob T -P

Rearrangement scenarios computed by CREx

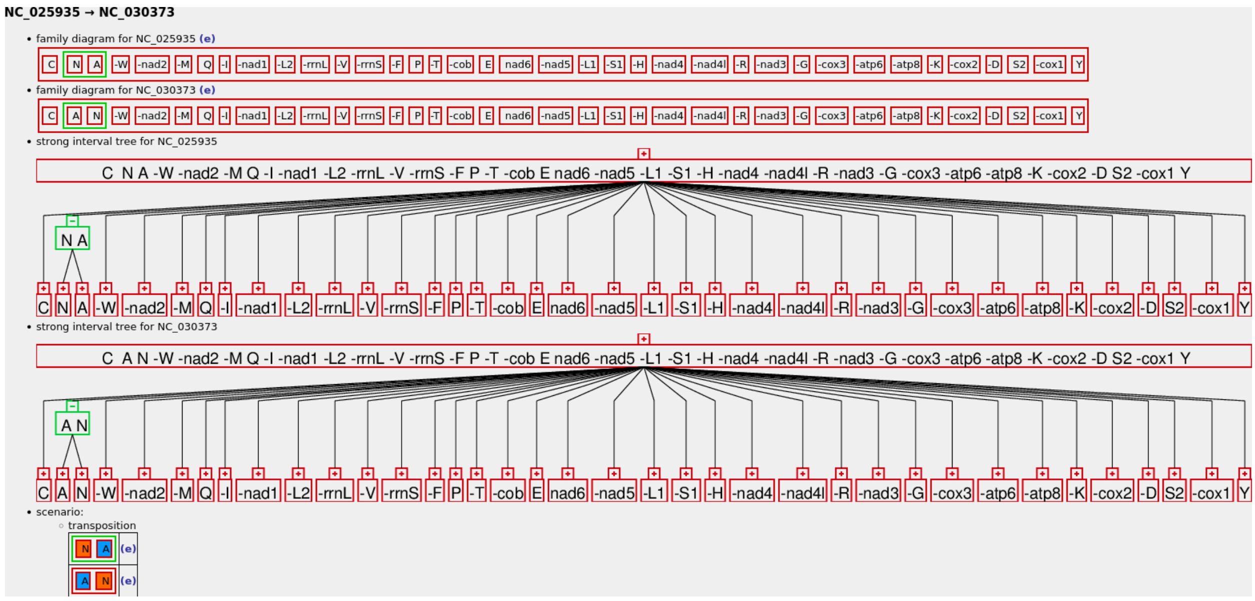

Figure S11: Rearrangement scenarios using common intervals in CREx [3] for first dislocation case study.

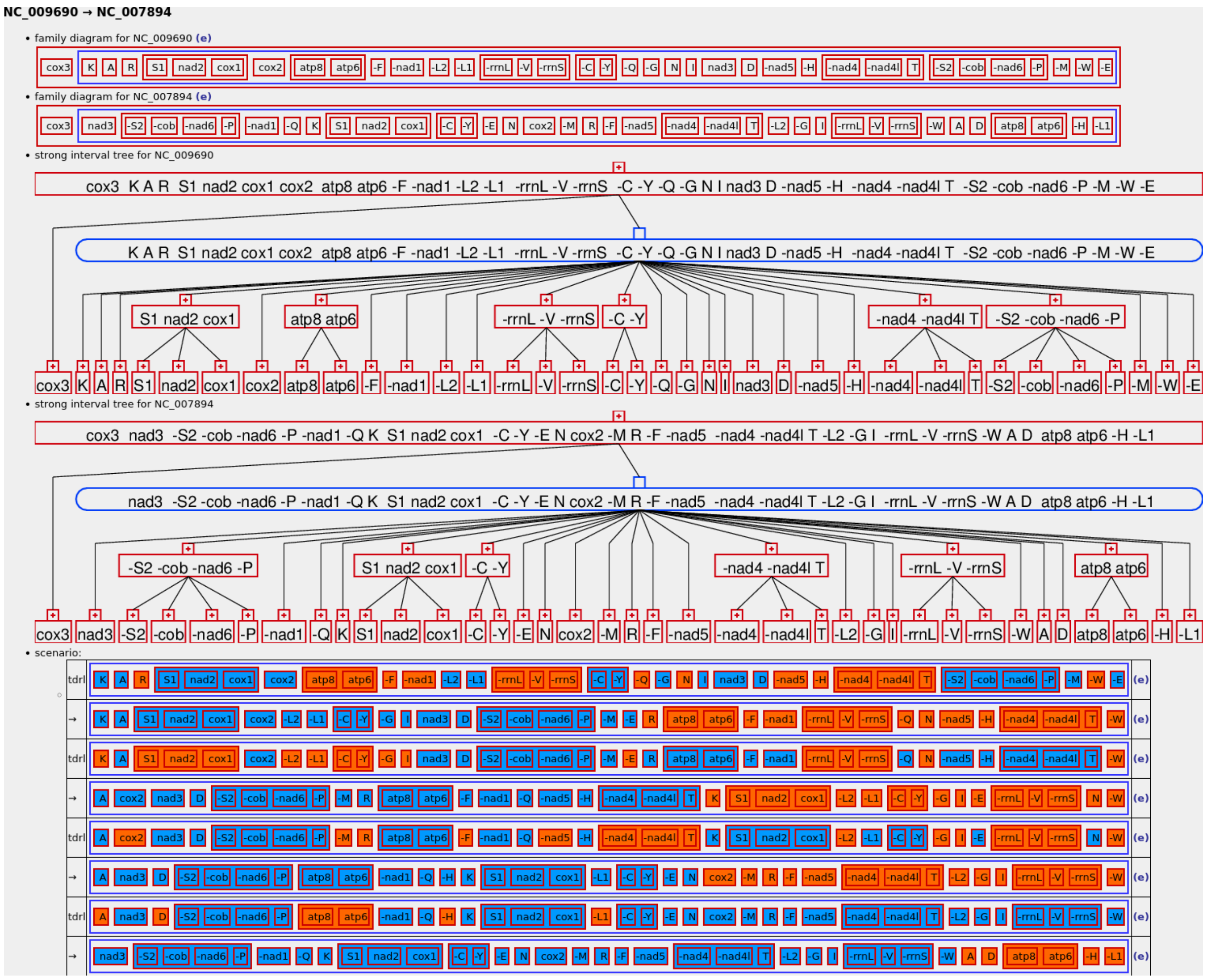

Figure S12: Rearrangement scenarios using common intervals in CREx [3] for second dislocation case study.

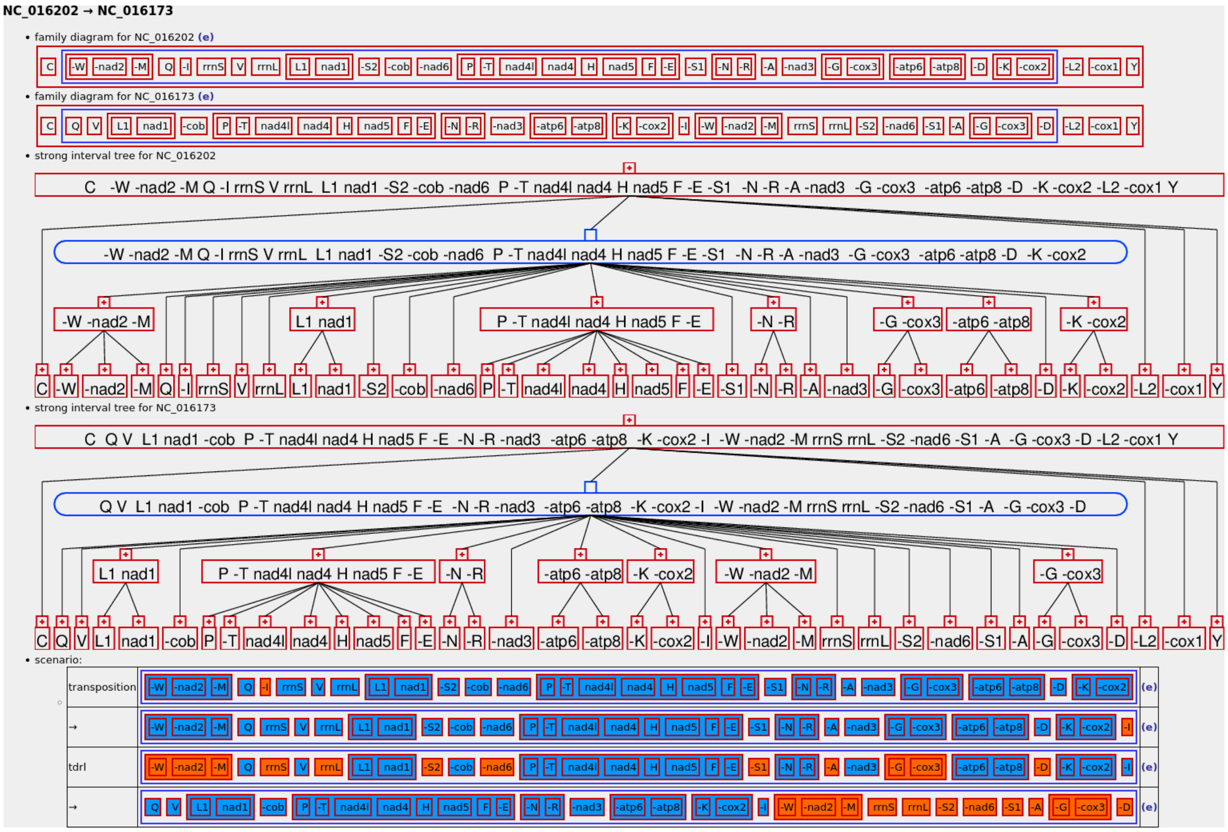

Figure S13: Rearrangement scenarios using common intervals in CREx [3] for third dislocation case study.

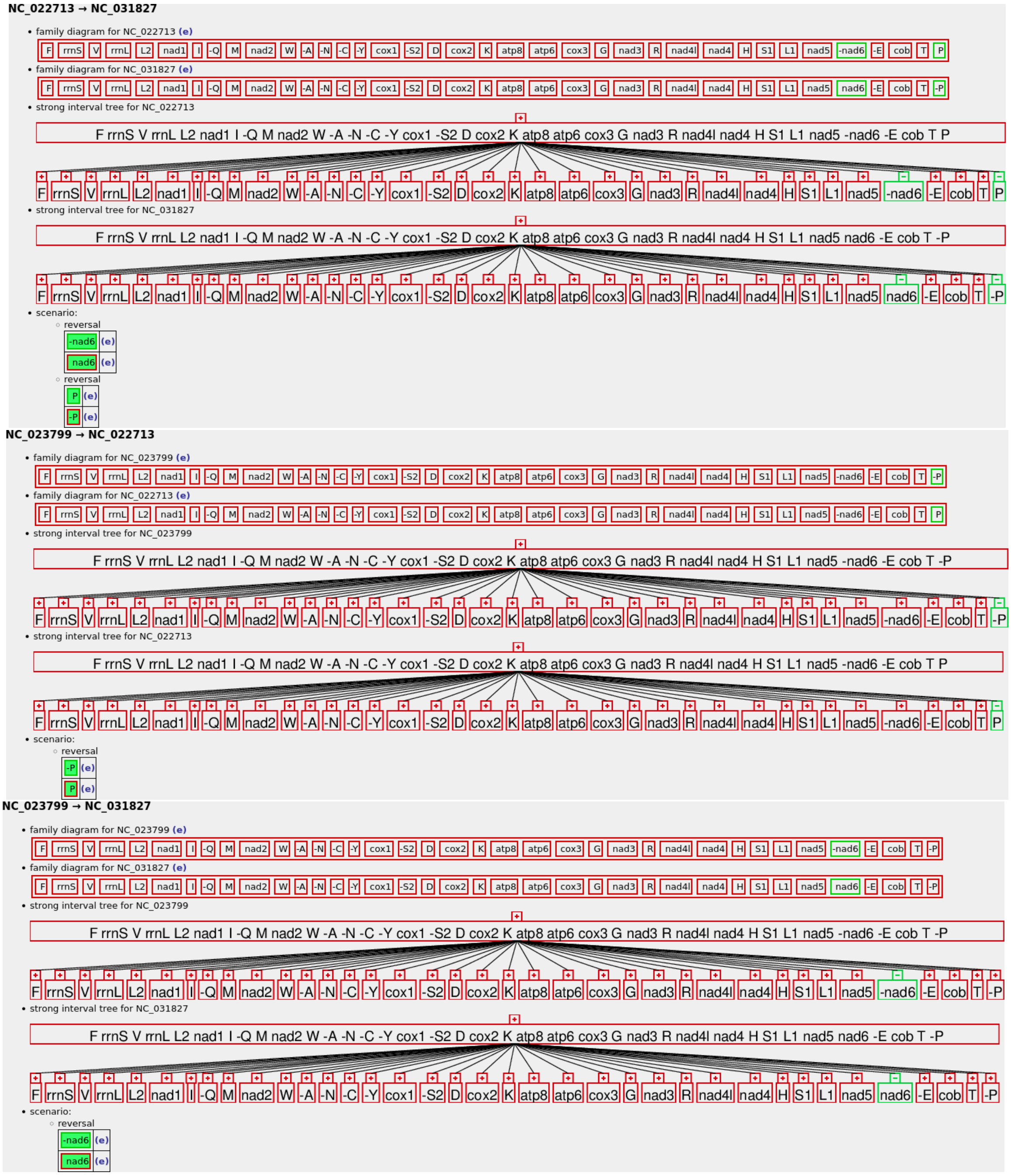

Figure S14: Rearrangement scenarios using common intervals in CREx [3] for inversion case study.

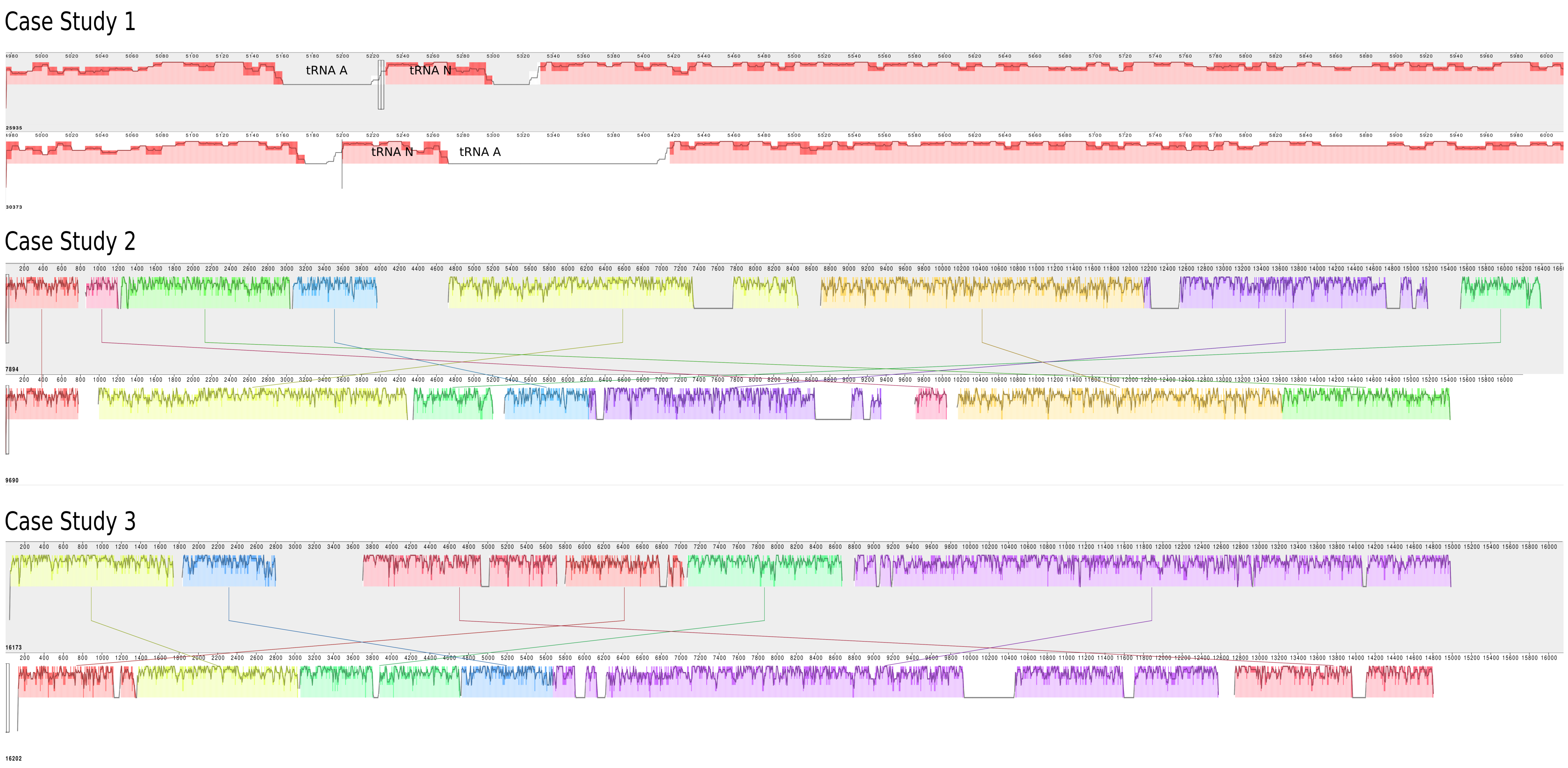

Figure S15: **progressiveMauve** alignments for the three dislocation case studies, generated by **progressiveMauve**'s viewing system. Case study 1: Only the relevant sequence segments are shown for the first case study. The breakpoints are missed in this case, as only tRNA N is aligned (annotated in the first plot). The aligned portions are indicated by a box in the first and vertical line in the second genome. Case study 2: Related collinear blocks are given the same color. All of the missed breakpoints are caused by long unaligned sequence segments. Case study 3: Missed breakpoints are caused by long unaligned sequence segments, or erroneously aligned segments. The latter causes two incorrect breakpoints, i.e., breakpoints that cannot be associated with any of the putative breakpoints.

Inversion case study

accession ID	inversed gene	start	end
NC_022713	tRNA-P	15589	15636
NC_23799		15602	15649
NC_23799	nad6	14054	14154
NC_031827		13952	14052
NC_022713	tRNA-P	15587	15647
NC_031827		15624	15684

Table S2: Inversion block locations predicted by `DeBBI`.

accession ID	inversed gene	start	end
NC_022713	tRNA-P	15584	15653
NC_23799		15585	15654
NC_23799	nad6	13777	14298
NC_031827		13808	14329
NC_022713	tRNA-P	15584	15653
NC_031827		15618	15687

Table S3: Putative inversion block locations annotated by `MITOS`.

Evaluation with Gecko and CHROMEISTER

For the real genome experiments (three dislocation case studies and one inversion case study, see main manuscript), we also evaluated the breakpoint predictions with two other sequence aligner `Gecko` and `CHROMEISTER`. To this end, we manually determined suitable parameter settings for each case study and tool. As outlined below, both tools produced predictions of poorer quality than `progressiveMauve` (and `DeBBI`) in all cases.

CHROMEISTER

In `CHROMEISTER`, the k-mer length needs to be specified, but only multiples of four are possible settings. We hence started with $k = 4$ and increased this value as long as more than one alignment block was contained in the output (if fewer alignment blocks are present, there are no breakpoints). Breakpoints could not be detected in any of the experiments. One explanation for this could be that the k-mer size can only be increased in multiples of four so that more suitable settings for our application case might get missed. `CHROMEISTER` hence seems to be a too coarse-grained approach for the detection of gene breakpoints in mitochondrial genomes.

Gecko

In `Gecko` the *relative alignment similarity*, which is the score attained by an alignment divided by the maximum possible score, must be provided. It must be specified as a percentage, i.e. can attain values between 0 and 100. Starting from 50, we subsequently increased this parameter by increments of 10 until the number of contradictory alignment blocks was at most 25% of the not-contradictory alignment blocks in order to keep the number of "random" alignments at bay. Thereby, contradictory alignments are such alignments where one sequence segment is aligned with multiple segments of the other sequence so that at most one of them is correct, whereas the others align unrelated sequence segments.

For case study 1, this resulted in a relative similarity score of 50, for case study 2 a value of 80, and for case study 3 a value of 70, and a value of 50 for the inversion case study. No breakpoints could be identified in the inversion and first dislocation case study. In each of case study 2 and 3, only two breakpoints could be identified with distances of at most 200 (cf. figs. S19 and S20).

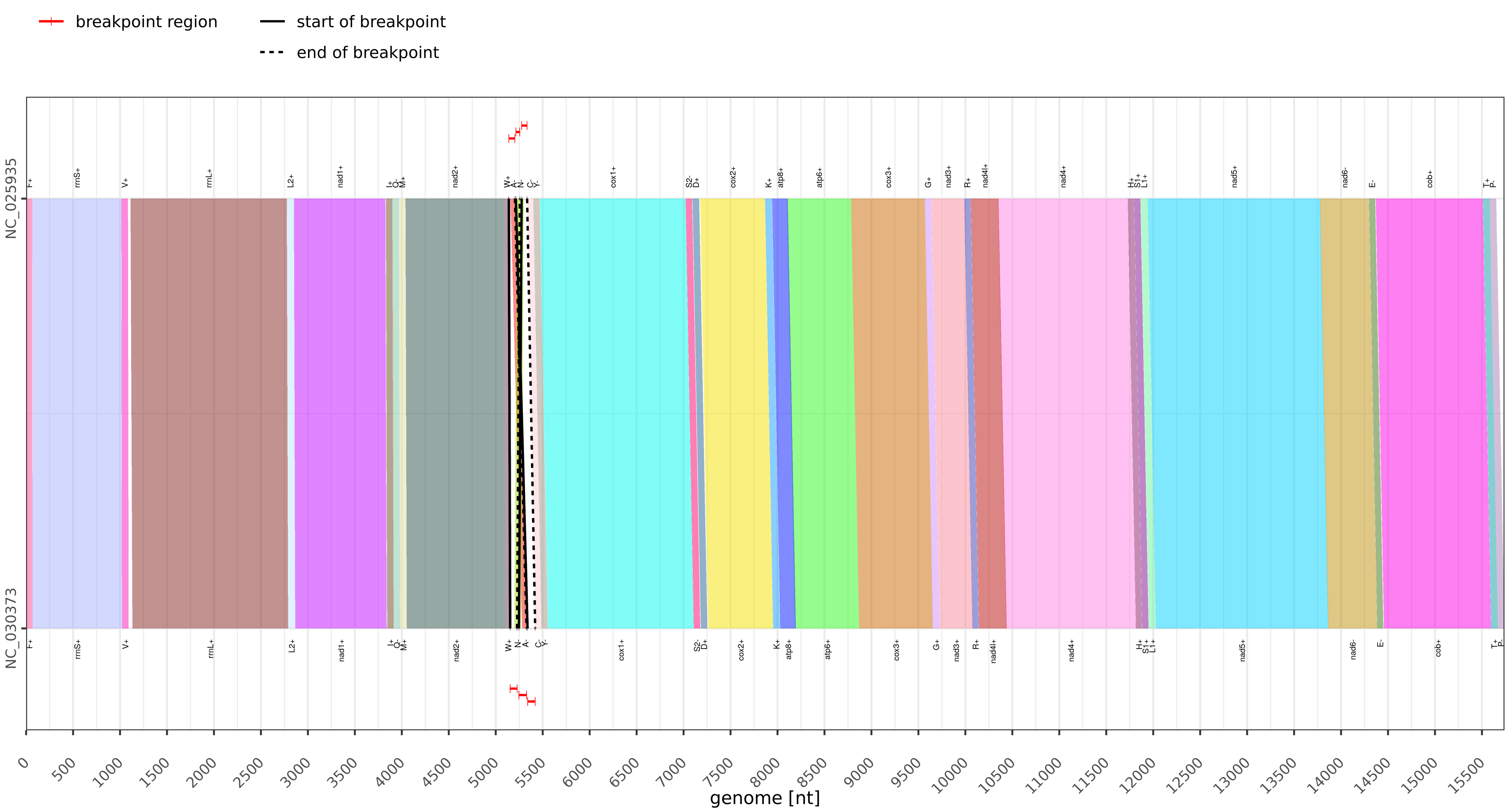

Figure S16: `DeBBI` breakpoint plot for first case study.

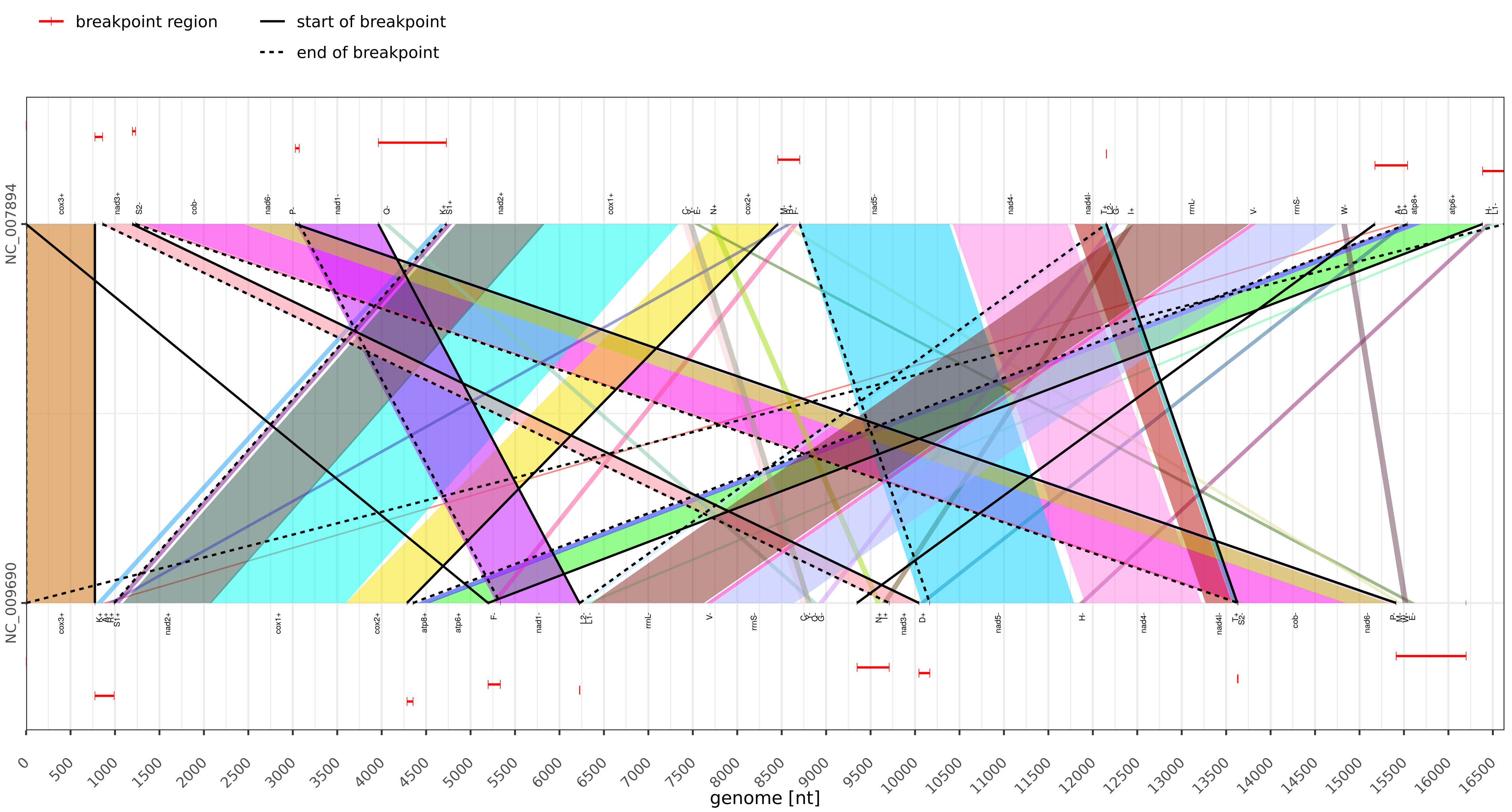

Figure S17: `progressiveMauve` breakpoint plot for second case study.

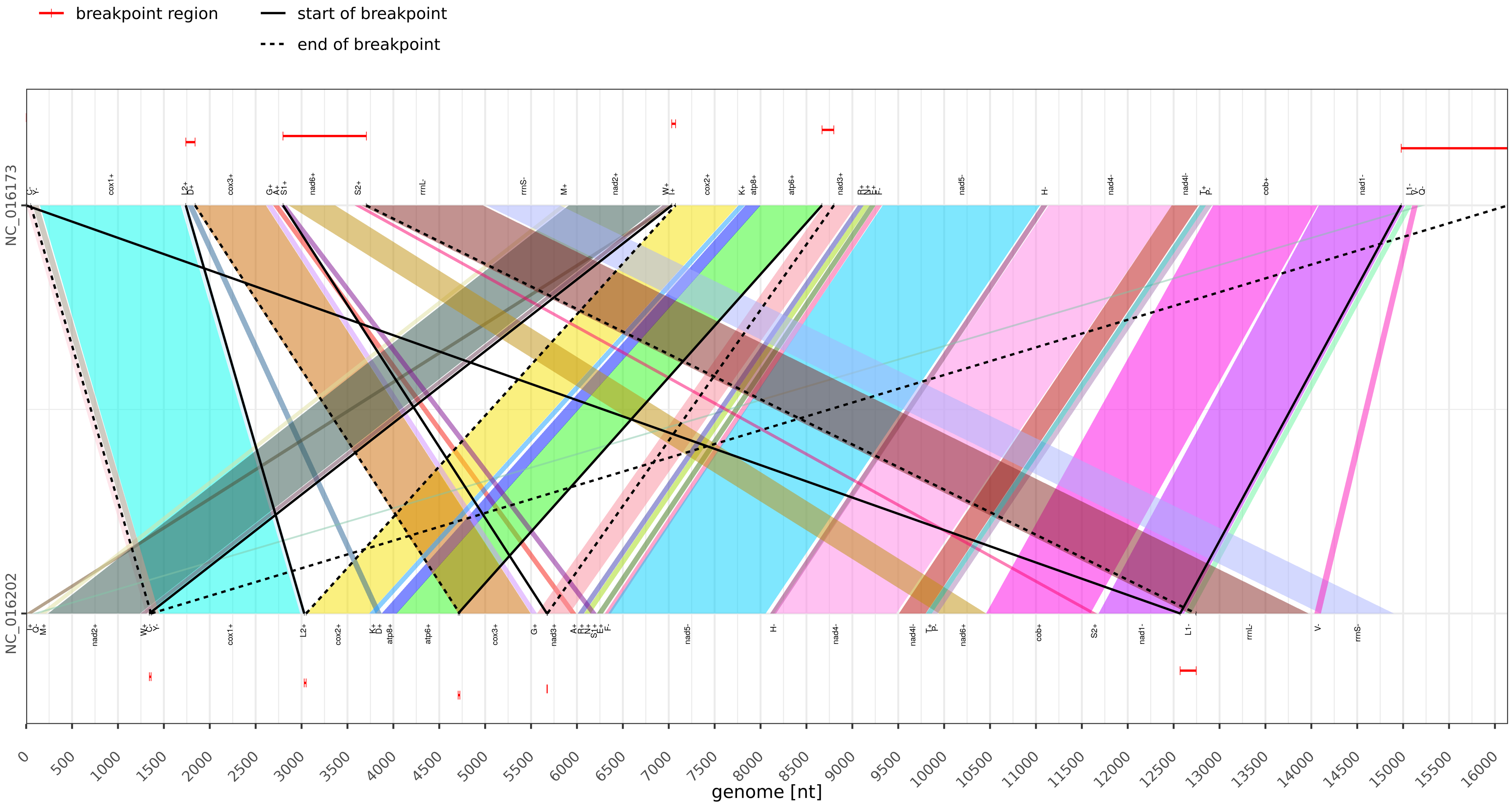

Figure S18: `progressiveMauve` breakpoint plot for third case study. `progressiveMauve` incorrectly aligns gene S1+ of the first with gene nad3+ of the second sequence. The line connecting the corresponding genes is anti-parallel to the colored gene blocks. This produces two incorrect breakpoint predictions.

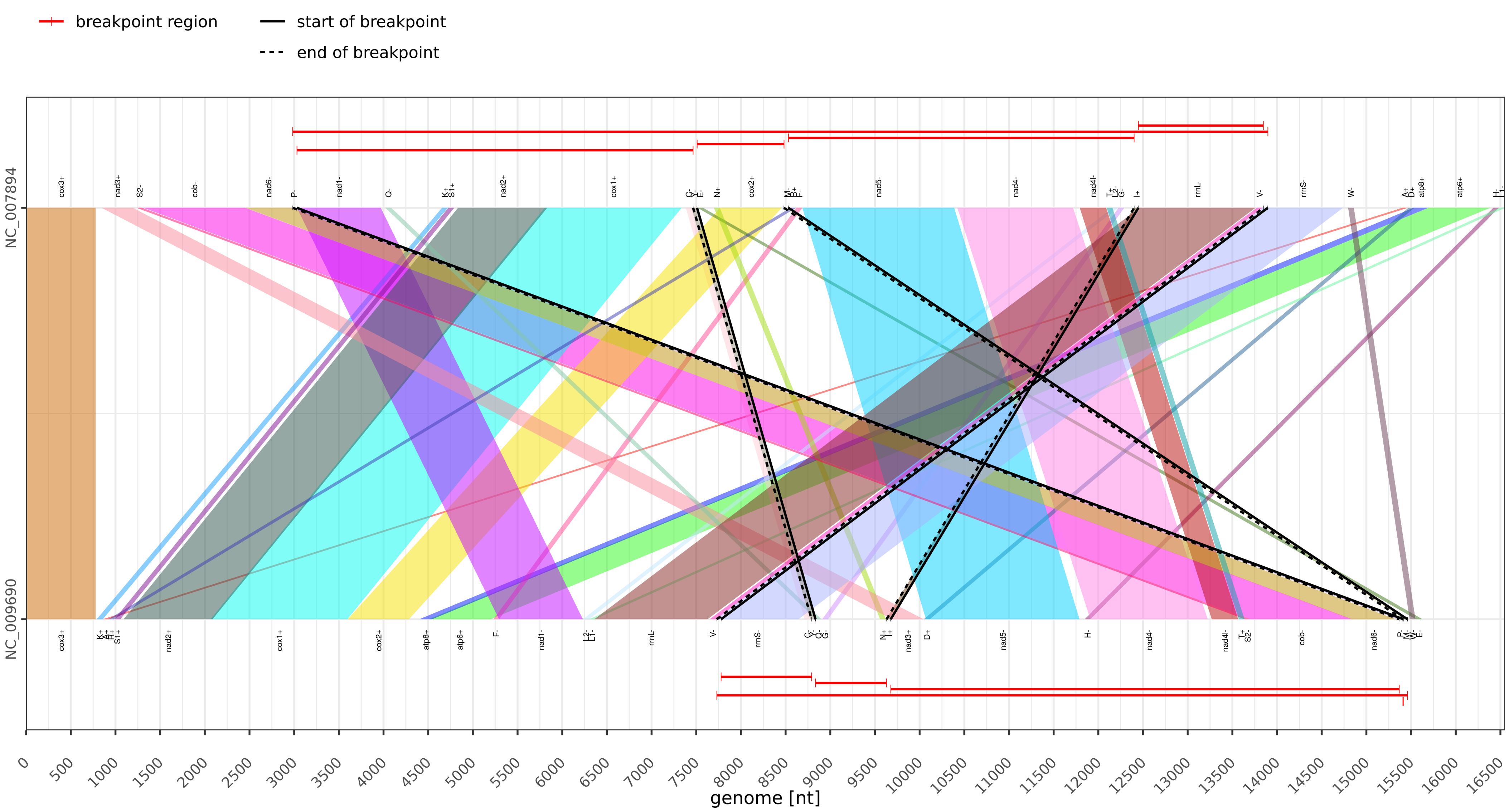

Figure S19: `Gecko` breakpoint plot for second case study. Only two breakpoints are identified with a distance of at most 200.

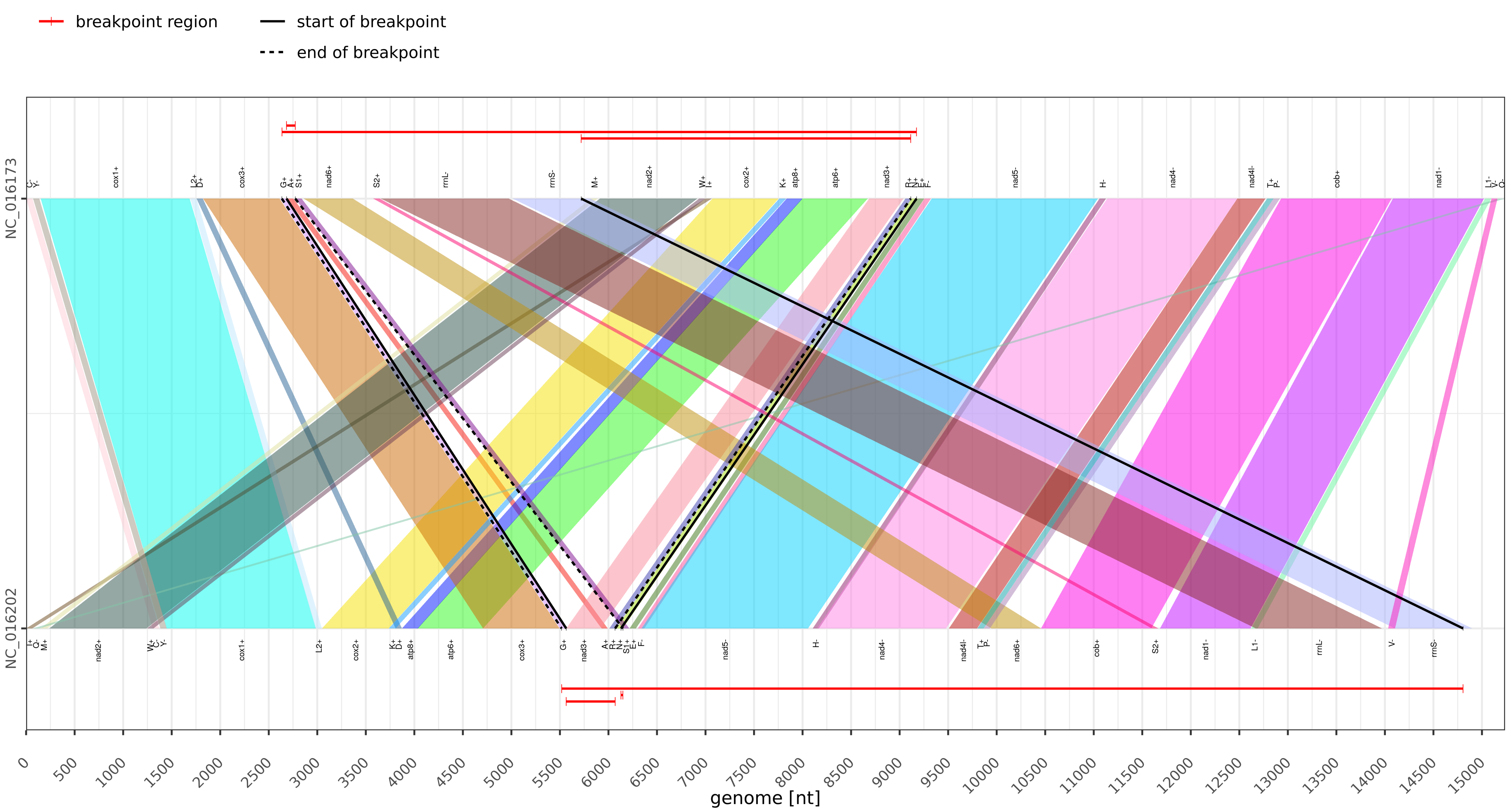

Figure S20: `Gecko` breakpoint plot for third case study. Only two breakpoints are identified with a distance of at most 200.

References

[1] Altschul SF, Gish W. Local alignment statistics. vol. 266 of Computer Methods for Macromolecular Sequence Analysis. Academic Press; 1996. p. 460–480.

[2] Lawless JF. Statistical Models and Methods for Lifetime Data. John Wiley & Sons; 2011.

[3] Bernt M, Merkle D, Ramsch K, Fritzsch G, Perseke M, Bernhard D, et al. CREx: inferring genomic rearrangements based on common intervals. Bioinformatics. 2007 Nov;23(21):2957–2958. Available from: https://doi.org/10.1093/bioinformatics/btm468.
